# Industrial‐current Ammonia Synthesis by Polarized Cuprous Cyanamide Coupled to Valorization of Glycerol at 4,000 mA cm^−2^

**DOI:** 10.1002/adma.202418451

**Published:** 2025-02-21

**Authors:** Jiacheng (Jayden) Wang, Huong T. D. Bui, Huashuai Hu, Shuyi Kong, Xunlu Wang, Hongbo Zhu, Junqing Ma, Jintao Xu, Yihong Liu, Lijia Liu, Wei Chen, Hui Bi, Minghui Yang, Fuqiang Huang, Tore Brinck, Jiacheng Wang

**Affiliations:** ^1^ The State Key Laboratory of High‐Performance Ceramics and Superfine Microstructure Shanghai Institute of Ceramics Chinese Academy of Sciences Shanghai 200050 China; ^2^ Center of Materials Science and Optoelectronics Engineering University of Chinese Academy of Sciences Beijing 100049 China; ^3^ Department of Chemistry CBH KTH Royal Institute of Technology Stockholm SE‐100 44 Sweden; ^4^ School of Environmental Science and Technology Dalian University of Technology Dalian 116024 China; ^5^ State Key Laboratory of Metal Matrix Composites School of Materials Science and Engineering Shanghai Jiao Tong University Shanghai 200240 China; ^6^ Department of Chemistry Western University 1151 Richmond Street London ON N6A5B7 Canada; ^7^ Department of Materials Design and Innovation University at Buffalo The State University of New York Buffalo NY 14260 USA; ^8^ Zhejiang Key Laboratory for Island Green Energy and New Materials Institute of Electrochemistry School of Materials Science and Engineering Taizhou University Taizhou 318000 China; ^9^ Key Laboratory of Advanced Energy Materials Chemistry (Ministry of Education) Nankai University Tianjin 300071 China

**Keywords:** ammonia synthesis, nitrate reduction, paired electro‐refinery system, surface electrostatic potential, techno‐economic analysis

## Abstract

The electrocatalytic nitrate reduction (NO_3_RR) holds significance in both NH_3_ synthesis and nitrate contamination remediation. However, achieving industrial‐scale current and high stability in membrane electrode assembly (MEA) electrolyzer remains challenging due to inherent high full‐cell voltage for sluggish NO_3_RR and water oxidation. Here, Cu_2_NCN with positive surface electrostatic potential *V*_S_(r) is applied as highly efficient NO_3_RR electrocatalysts to achieve industrial‐current and low‐voltage stable NH_3_ production in MEA electrolyzer with coupled anodic glycerol oxidation. This paired electro‐refinery (PER) system reaches 4000 mA cm^−2^ at 2.52 V and remains stable at industrial‐level 1000 mA cm^−2^ for 100 h with the NH_3_ production rate of 97000 µg_NH3_ h^−1^ cm^−2^ and a Faradaic efficiency of 83%. Theoretical calculations elucidate that the asymmetric and electron‐withdrawing [N−C≡N] units enhance polarization and V*_S_* (r), promoting robust and asymmetric adsorption of NO_3_^*^ on Cu_2_NCN to facilitate O−N bond dissociation. A comprehensive techno‐economic analysis demonstrates the profitability and commercial viability of this coupled system. Our work opens a new avenue and marks a significant advancement in MEA systems for industrial NH_3_ synthesis.

## Graphical abstract

This work presents a membrane electrode assembly (MEA) system featuring Cu_2_NCN as an efficient nitrate reduction (NO_3_RR) electrocatalyst, coupled with glycerol oxidation for low‐voltage, industrial‐level NH_3_ production. Achieving 4000 mA cm^−2^ at 2.52 V with sustained stability at 1000 mA cm^−2^ over 100 h, the system offers 83% Faradaic efficiency, with theoretical insights on NO₃^*^ adsorption and O–N bond cleavage.

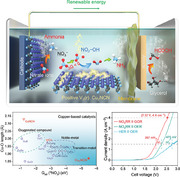

## 1 Introduction

Ammonia (NH_3_) and nitrate (NO_3_^−^) are very important N‐containing compounds that are strongly related to human activities. NH_3_ exhibits great potential as a hydrogen‐rich fuel for future generations and is an essential resource for nitrogen in both industrial and agricultural production.^[^ ^1^ ^]^ However, industrial‐scale NH_3_ synthesis relies heavily on the Haber–Bosch process, which not only requires high temperature (≈500 °C) and high pressure (>100 atm), but also releases 400 Mt of CO_2_ every year.^[^ ^2^ ^]^ NO_3_^−^ is enriched in industrial wastewater and contaminated groundwater, endangering human health and causing an imbalance in the nitrogen cycle. The direct electrochemical reduction of NO_3_^−^ (NO_3_RR) to NH_3_, driven by electricity generated from renewable energy, has been proposed as a clean and sustainable alternative to the above denitrification and subsequent Haber–Bosch process. It could enable NH_3_ synthesis under ambient conditions while offering potential environmental restoration and energy benefits.^[^ ^3^ ^]^ However, its industrial application suffers from small current density of <100.0 mA cm^−2^ and low NH_3_ yield due to the sluggish rate‐determining adsorption of NO_3_^*^ and the limitation of reaction devices, such as cylindrical and *H*‐type cells based on the three‐electrode system. It is really far from real industrial conditions, leading to the difficulty in calculating energy consumption.

The membrane electrode assembly (MEA) electrolyzer is considered as the most promising reactor with the advantages of low ohmic resistance and ease of scaling up to multicell stacks,^[^ ^4^ ^]^ but the high energy barrier of NO_3_^−^ activation and eight‐electron transfer steps hinders the realization of industrial‐scale high currents and high stability in MEA. Furthermore, the cathodic NO_3_RR typically coupled with the anodic oxidation evolution reaction (OER) requires inherent high full‐cell voltage due to the high thermodynamic potential for OER of 1.23 V vs. the reversible hydrogen electrode (RHE). The glycerol oxidation reaction (GOR) with an electrodynamic potential (E^θ^ = 0.69 V vs. RHE, Note S1, Supporting Information) is more thermodynamically favorable than the OER.^[^ ^5^ ^]^ Moreover, the industrial MEA should be achieved with a low full voltage, because high voltage increases the competition of hydrogenolysis, production cost, and energy requirement. Thus, developing advanced energy conservation and consumption reduction strategy for efficient NH_3_ electrolysis in MEA electrolyzer is still of great challenge but highly desirable.

**Scheme 1:**
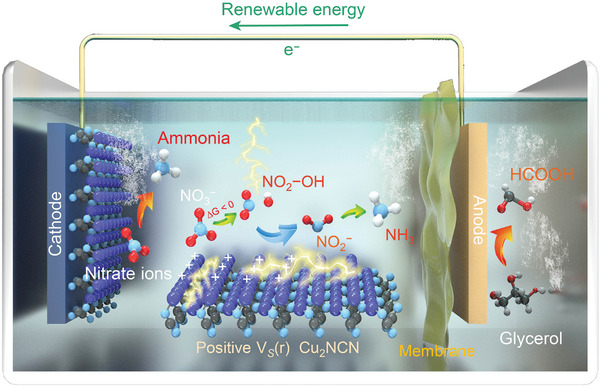
Schematic illustration of constructing a paired electro–refinery (PER) for co‐production of NH_3_ and formic acid powered by renewable energy. The membrane–electrode assembly (MEA)–based electrolyzer with surface positive V*_s_* (r) Cu_2_NCN as the cathode for the NO_3_RR coupling NiCo_2_O_4_ as the anode for the glycerol oxidation reaction (GOR) exhibits simultaneous NH_3_ production and glycerol valorization at industrial‐scale currents.

Herein, we report cuprous cyanamide (Cu_2_NCN) with positive surface electrostatic potential *V*_S_(r) as a highly efficient NO_3_RR electrocatalyst in the paired electro‐refinery (PER) MEA electrolyzer coupling with the GOR, exhibiting industrial currents and low voltage under ambient conditions (**Scheme** **1**). Theoretical calculations elucidate that the asymmetric and electron‐withdrawing [N−C≡N] units in Cu_2_NCN enhanced polarization and positive surface electrostatic potential *V*_S_(r), promoting robust and asymmetric adsorption of NO_3_^*^, facilitating O−N bond dissociation, and accelerating hydrogenation. Our PER system reaches 4,000 mA cm^−2^ at 2.52 V and remains stable at an industrial‐level current of 1,000 mA cm^−2^ for 100 h with the NH_3_ production rate of 97,000 µg_NH3_ h^−1^ cm^−2^ and a Faradaic efficiency of 83%. Comprehensive techno‐economic analysis (TEA) shows the economic viability of our lab‐made system incorporating NO_3_RR and GOR for paired electrosynthesis of green ammonia and high‐value anodic products. Our work opens a new avenue and marks a significant advancement in MEA reactors for industrial NH_3_ synthesis in the PER system.

## 2 Results and Discussion

### 2.1 Screening Cu‐Based NO_3_RR Catalysts Applied in the PER

**Figure 1:**
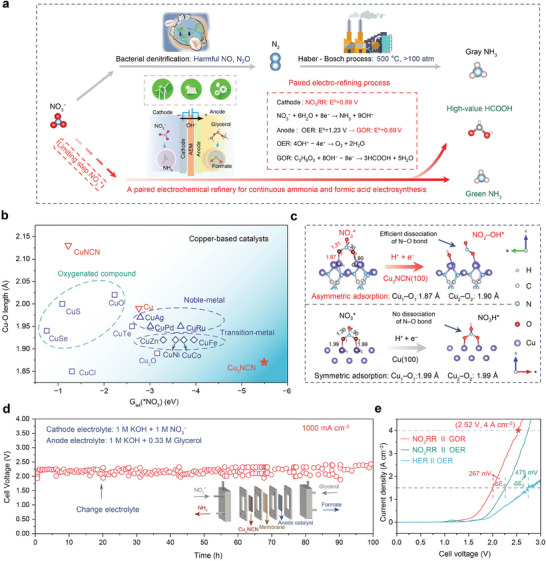
Comparing two kinds of nitrogen cycles and screening Cu‐based NO_3_RR catalysts applied in the PER for industrial continuous electrosynthesis of ammonia and formic acid. a) The conventional nitrogen cycle (gray arrows) based on bacterial denitrification and Haber–Bosch process, and the direct electrocatalytic nitrogen cycle (red arrows) involving NO_3_^−^ reduction to NH_3_ using renewable and intermittent electricity. Inset: Schematic of constructing a PER with Cu_2_NCN as the cathode for the NO_3_RR and NiCo_2_O_4_ as the anode for the GOR, showing the simultaneous production of NH_3_ and formic acid. b) Screening of Cu‐based electrocatalysts. All the catalysts are compared at the adsorption energies of NO_3_^*^ and the Cu−O bond lengths, among which polarized Cu_2_NCN shows the largest adsorption energy of NO_3_^−^ with the shortest Cu−O bond length. c) The asymmetric and strong adsorption leads to smooth dissociation of the O−N bond in NO_3_^*^ on Cu_2_NCN(100), thus forming NO_2_−OH^*^ over hydrogenation during the NO_3_RR process. In sharp contrast, the symmetric adsorption of NO_3_^*^ on Cu or CuNCN facilitates the formation of NO_3_H^*^ over hydrogenation with increased difficulty in the O−N bond breaking (Figure S1, Supporting Information). d) Continuous tests of the Cu_2_NCN catalyst at industrial 1000 mA cm^−2^ for 100 h using a MEA‐based electrolyzer for the PER process. e) LSV curves using the PER with and without glycerol at the anode. The OER//HER LSV curve is also shown for comparison.

The conventional nitrogen cycles involve transferring NO_3_^−^ contaminants to N_2_ by bacterial denitrification and the subsequent Haber−Bosch process for ammonia synthesis (the route indicated by gray arrows in **Figure** **1**a). In sharp contrast, the direct electrochemical reduction of NO_3_^−^ (NO_3_RR) to NH_3_, driven by electricity generated from renewable energy (the route indicated by red arrows in Figure 1a), is a clean and sustainable alternative to the above denitrification and subsequent Haber–Bosch process. The paired electro‐refining process toward cathodic NO_3_RR coupled with anodic GOR (E^θ^ = 0.69 V vs. RHE) instead of OER (E^θ^ = 1.23 V vs. RHE) shows lower energy consumption and efficient generation of double high‐value products NH_3_ and formate.

By calculating the adsorption energies and Cu−O bond lengths of ^*^NO_3_ adsorbed onto the surfaces of various Cu‐based electrocatalysts (Figure 1b), we observe that polarized Cu_2_NCN exhibits the greatest adsorption energy of NO_3_^−^ and the shortest Cu−O bond length. This indicates the strongest interaction between NO_3_^−^ and Cu_2_NCN, favorable for N−O bond dissociation during the NO_3_RR process. Notably, the polarization of Cu_2_NCN leads to a stronger interaction with NO_3_^*^ and a significant reduction in the Cu−O bond lengths (1.87 and 1.90 Å) and a slight elongation of the O−N bond length (1.31 and 1.30 Å) (Figure 1c and Figure S1, Supporting Information) compared to the Cu (Cu−O 1.99 Å, O−N 1.30 Å) and CuNCN (Cu−O 2.12 Å, O−N 1.29 Å) configurations (Figures S1,S2, Supporting Information). This strong and asymmetric adsorption facilitates the smooth O−N bond dissociation of NO_3_^*^ over hydrogenation on Cu_2_NCN, resulting in the formation of NO_2_−OH^*^ and a subsequent boost in the NO_3_RR process (Figure 1c). In contrast, the symmetric and weaker adsorption of NO_3_^*^ on nonpolar Cu or CuNCN fosters the preferential formation of NO_3_H^*^ over hydrogenation, thereby making the cleavage of O−N bonds more difficult. Additional theoretical calculations indicate that the electron transfer from polarized Cu_2_NCN (100) to NO_3_^*^ is significantly enhanced compared with those of NO_3_^*^ on Cu (100) and CuNCN (100) (Figure S2, Supporting Information).

To investigate practical applications of polarized Cu_2_NCN for ammonia synthesis, we constructed a paired electro‐refinery (PER) by coupling cathodic NO_3_RR and anodic glycerol oxidation reaction (GOR) using membrane–electrode assembly (MEA)‐based electrolyzer (the inset in Figure 1d). Our paired electro‐refinery reached 4,000 mA cm^−2^ at 2.52 V (Figure 1e) and remained stable at industrial‐level 1,000 mA cm^−2^ for 100 h. This result suggests industrial prospects of the paired valorization route with polarized Cu_2_NCN for the NO_3_RR.

### 2.2 Surface Electrostatic Potential and Structure Characterization of Polarized Cu_2_NCN

**Figure 2:**
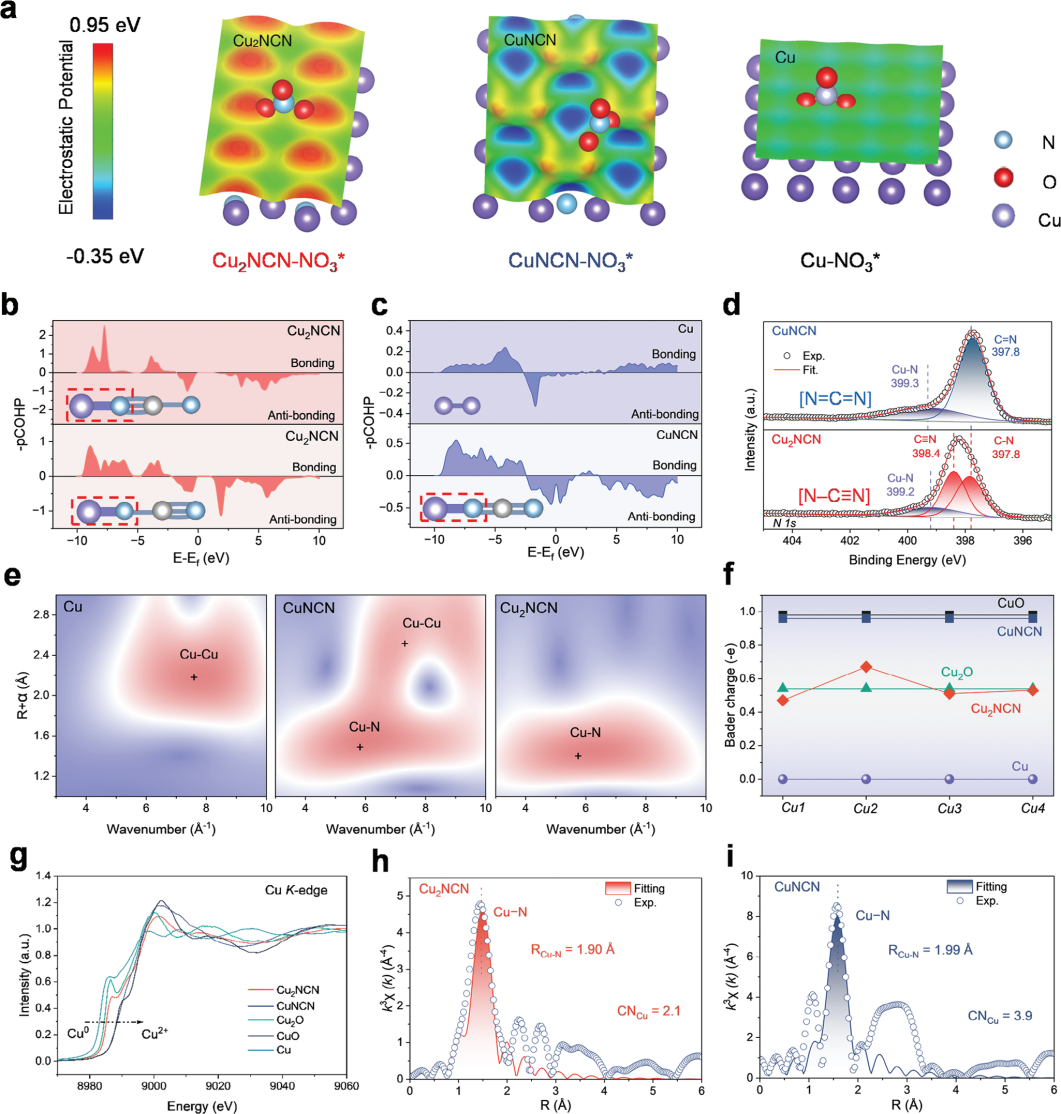
Structure characterization of polarized Cu_2_NCN. a) The surface electrostatic potential (*V*_S_(r)) at the 0.001 au isodensity surface of the bare Cu_2_NCN, CuNCN and Cu structures. In each structure, NO_3_^–^ has been placed at the atomic positions from the DFT optimization of NO_3_^–^ adsorption to indicate the preferred binding mode. It can be seen that for each structure, NO_3_^–^ binds at the position above Cu where it achieves the most favorable electrostatic interaction with the surface. The polarized Cu_2_NCN has a *V*_S_(r) that is everywhere positive with high valued maxima at the O binding positions above the protruding Cu atoms, and thus provides an ideal binding site for NO_3_^−^. The CuNCN surface has negative areas (blue) above the protruding N that leads to a less favored electrostatic interaction when NO_3_^−^ binds to the Cu atom at the positive surface ridge between the N minima. The Cu surface provides similar binding sites at the positive Cu atoms as Cu_2_NCN, but the *V*_S_(r) is lower at these sites and thus it binds NO_3_^–^ weaker. b, The COHP of the Cu–N bond in Cu_2_NCN, exhibiting two kinds of Cu coordination environments. Inset: schematic models of Cu–[N≡C–N] and Cu–[N–C≡N] existing in Cu_2_NCN. c) The COHP of the Cu–Cu and Cu–N bonds in Cu and CuNCN, respectively. It only shows one coordination environment for Cu sites in Cu and CuNCN. Inset: schematic models of Cu–Cu and Cu–[N=C=N]^2–^ in Cu and CuNCN, respectively. d) N 1S XPS spectra of CuNCN and Cu_2_NCN. e) Wavelet‐transformed (W. T.) EXAFS patterns at Cu *K*‐edge with the optimized Morlet parameter for Cu, CuNCN and Cu_2_NCN at the first coordination shell. f) Bader charge distribution of Cu, CuO, Cu_2_O, CuNCN, and Cu_2_NCN. g) Cu *K*‐edge XANES spectra for Cu, CuNCN and Cu_2_NCN. h,i) Fourier‐transformed EXAFS and corresponding fitting curves of (h) Cu_2_NCN and (i) CuNCN at the first coordination shell.

Our previous work shows that the binding strength of Lewis bases, such as NO_3_^−^, to materials based on the coinage metals, i.e. Cu, Ag, and Au, correlates with the magnitude of the positive *V*_S_(r) at the metal binding site.^[^ ^6^ ^]^ This type of interaction is a consequence of σ‐holes, i.e. electron deficient areas with positive surface electrostatic potential *V*_S_(r), at the low‐coordinated and polarized metal sites.^[^ ^6b^ ^]^ Herein, the polarized Cu_2_NCN, and typical references Cu and CuNCN were selected for *V*_S_(r) analysis. The Cu_2_NCN surface has protruding Cu atoms that are positively charged because of their bonding to the electron‐withdrawing [N−C≡N] units (**Figures** **2**a and S3, Supporting Information), and this leads to a net surface polarization, as indicated by a DFT computed dipole moment of 0.46 electrons•Å per simulation cell. More importantly, *V*_S_(r) is everywhere positive with maxima at the protruding Cu atoms. Furthermore, the distance between the positive Cu is suitable for binding bidentate NO_3_^–^ to two separate Cu, i.e. one O binding to each Cu. In contrast to the Cu_2_NCN surface, the CuNCN surface has slightly protruding N atoms of negative charge, and the slightly positive Cu binding sites are located at the ridges between the N. This structure has no net polarization, and *V*_S_(r) varies between positive and negative values (Figure 2a). For pure Cu, its surface is nonpolar with only a small variation in *V*_S_(r), and the *V*_S_(r) maxima on the Cu surface are much less positive than those of Cu_2_NCN.

The as‐synthesized Cu_2_NCN catalyst is composed of nanosheets with the strongest diffraction of the (100) plane (Figure S4, Supporting Information). The Cu−N coupling effect in Cu_2_NCN and CuNCN was analyzed by calculating the crystal orbital Hamilton population (COHP). The negative and positive values of −COHP correspond to the anti‐bonding and bonding state, respectively. Cu_2_NCN is built up by [N≡C−N] units which are coordinated to Cu as Cu[N≡C−N]Cu_3_. Each first layer N (i.e. N−C) is bonded to three Cu, one from the top layer Cu and two from the second layer Cu. In the next layer of N (i.e. N≡C), each N is coordinated to a single Cu. This alternating structure motif is then reversed and continued throughout the Cu_2_NCN crystal structure as Cu_3_[N−C≡Ν]Cu[N≡C−N]Cu_3_. The alternation between N≡C and N−C bonds is further evidenced by the alternating N−C bond lengths, of 1.30 and 1.19 Å for the first NCN unit and 1.20 and 1.27 Å in the second unit (Figure 2b). In contrast, only one state of Cu coupling exists in pure Cu and CuNCN (i.e., Cu−Cu and Cu−[N═C≡N]) (Figure 2c). It indicates an asymmetrical and surface‐polarized crystal structure of Cu_2_NCN. The characteristic vibration peaks in the Infrared (IR) spectra also clearly reveal the existence of asymmetrical [N−C≡N]^2−^ anions in the Cu_2_NCN structure (Figure S5, Supporting Information), significantly different from CuNCN with its symmetrical [N═C≡N]^2−^ anions. The deconvoluted X‐ray photoelectron spectroscopy (XPS) spectra (Figure 2d) and the Auger electron spectrum of Cu_2_NCN (Figure S6, Supporting Information) indicate that Cu(I) is the major surface species and that it binds to the N end of [N−C≡N]^2–^ anion.^[^ ^7^ ^]^ This allows for asymmetric adsorption of NO_x_^*^ intermediates, and it is thus beneficial for facilitating the hydrogenation of NO_x_^*^ and O−N bond dissociation in NO_3_RR.

More detailed information about the Cu electronic state and local binding environment in Cu_2_NCN was deduced from the X‐ray absorption near‐edge structure (XANES) and the extended X‐ray absorption fine structure (EXAFS). Moreover, the *k*‐space information was revealed by the wavelet‐transformed EXAFS spectra (Figure 2e), in which Cu–N region (R_Cu–N_: ≈1.45 Å, k_Cu–N_: ≈6.5 Å^−1^) was found in Cu_2_NCN in the first coordination shell. The lower R_Cu–N_ value for Cu_2_NCN suggests a stronger Cu−N interatomic interaction compared to CuNCN (R_Cu–N_: ≈1.6 Å), also indicating longer Cu−Cu coordination distance different from pure Cu (R_Cu–Cu_: ≈2.3 Å) and CuNCN (R_Cu–Cu_: ≈2.6 Å).^[^ ^8^ ^]^ This is ascribed to the asymmetrical dispersion of electron states in Cu_2_NCN. Furthermore, the Bader charge distribution also confirms the asymmetrical Cu sites. Compared to Cu, CuO, Cu_2_O, and CuNCN, Cu_2_NCN displays different valence states (Figure 2f and Figure S7, Supporting Information) and asymmetrical electron density states around Cu atoms (Figure S8, Supporting Information), indicating enhanced polarization in Cu_2_NCN. The XANES spectra at Cu *K*‐edge reveal that the energy absorption threshold of Cu_2_NCN is located between those of Cu and CuNCN (Figure 2g), suggesting that the valence state of Cu_2_NCN is situated at Cu^+^. Further fitting of the Fourier‐transformed EXAFS spectra illustrates that Cu−N distances are ≈≈1.90 Å (Cu_2_NCN) and 1.99 Å (CuNCN), respectively (Figure 2h,i). The overall coordination number (CN) of Cu_2_NCN in the first shell is 2.1 (Table S1, Supporting Information), substantially lower than that in CuNCN (CN = 3.9), revealing the undercoordinated states of Cu due to the asymmetric [N−C≡N]^2−^ binding.

### 2.3 Electrocatalytic NO_3_RR Evolution of Polarized Cu_2_NCN

**Figure 3:**
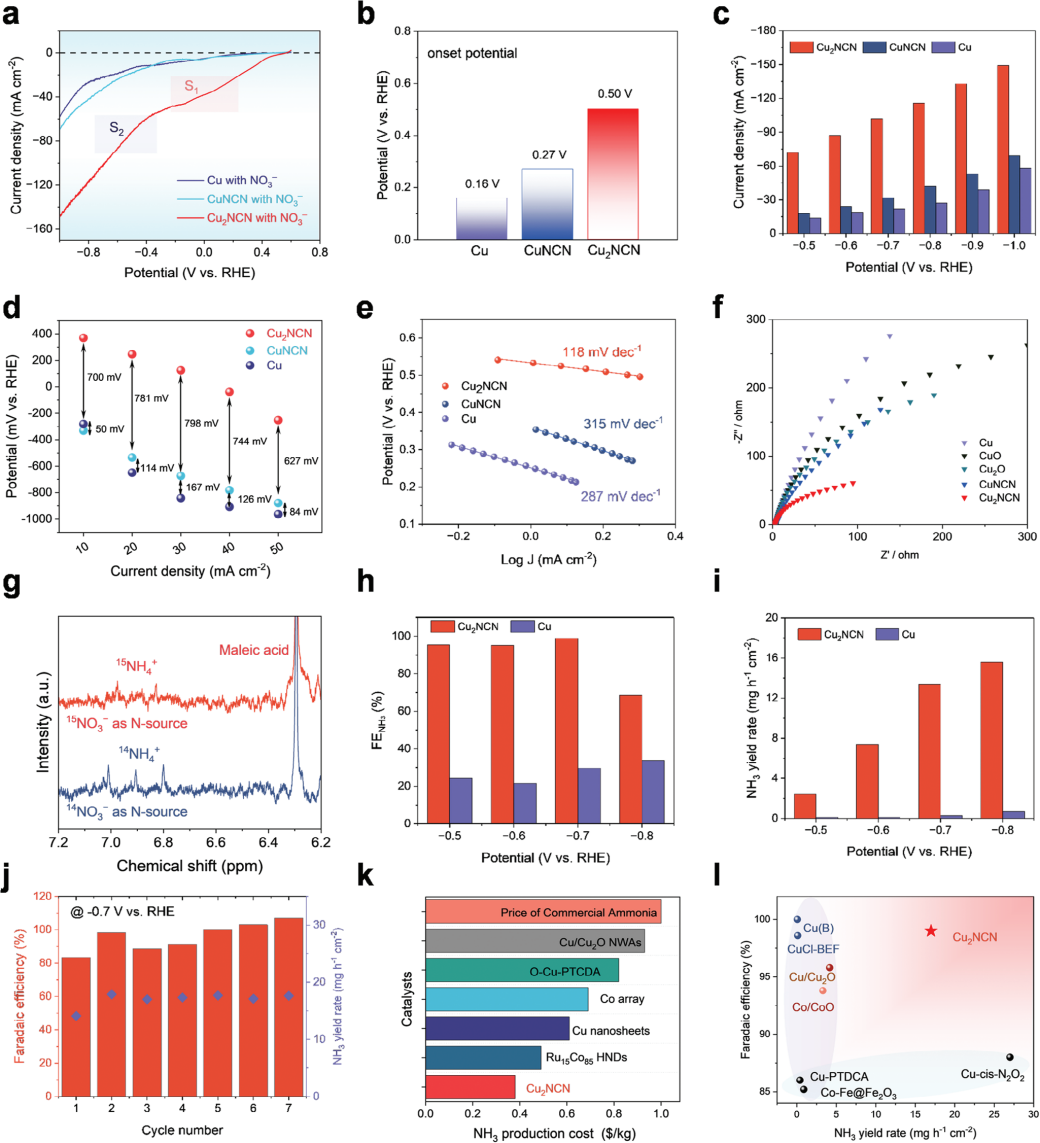
Electrocatalytic performance of NO_3_RR by polarized Cu_2_NCN. a) LSV curves of Cu_2_NCN, CuNCN, and Cu in a neutral electrolyte containing 500 ppm KNO_3_. b) Electrochemical overpotentials (vs. RHE) at −20 mA cm^−2^ for Cu, CuNCN, and Cu_2_NCN. c) NO_3_RR current densities of Cu_2_NCN, CuNCN, and Cu at different overpotentials. d) NO_3_RR overpotentials of Cu_2_NCN, CuNCN, and Cu vs. RHE at different current densities. e,f) The Tafel plots (e), and Electrochemical Impedance Spectroscopy (f) of Cu, CuNCN, and Cu_2_NCN. g) ^1^H NMR spectra of produced NH_3_ from ^15^NO_3_^−^ and ^14^NO_3_^−^ feeding. h,i) NH_3_ Faradaic efficiency (h) and NH_3_ yields (i) for Cu and Cu_2_NCN at the given potentials. j) The cyclic stability test and corresponding NH_3_ Faradaic efficiency and yields of Cu_2_NCN. k) Comparison of the NH_3_ production cost by NO_3_RR using Cu_2_NCN and the recently reported catalysts. l) The performance comparison of polarized Cu_2_NCN with the previously reported electrocatalysts toward NO_3_RR under neutral low‐concertation (<1000 ppm) nitrate solutions.

To determine the onset potential of NO_3_RR, a linear sweep voltammetry test of the Cu_2_NCN electrode was first performed in a neutral electrolyte containing 0.5 m K_2_SO_4_ and 500 ppm KNO_3_−N at 25 °C (**Figure** **3**a and details in supporting methods). The results imply that the Cu_2_NCN catalyst exhibits a lower onset potential of 0.5 V versus RHE than Cu (0.16 V), CuNCN (0.27 V), and other reported electrocatalysts (Figure 3b and Table S2, Supporting Information). At all investigated overpotentials, Cu_2_NCN exhibits a larger current density than Cu and CuNCN, suggesting a higher NO_3_RR activity of Cu_2_NCN (Figure 3c,d). We also compare the electrocatalytic activity toward the HER and NO_3_RR. The obvious decrease of current densities in the absence of NO_3_^–^ suggests low HER activity (Figure S9, Supporting Information). The Tafel slopes, findings from Electrochemical Impedance Spectroscopy (EIS), and calculated double‐layer capacitance all indicate a faster charge transfer rate for Cu_2_NCN (Figure 3e,f and Figure S10, Supporting Information). ^15^N isotope labeling experiments were conducted to further verify that the produced NH_3_ was resulting from the feeding NO_3_^−^ electrolyte.^[^ ^9^ ^]^ After electrolysis at −0.7 V versus RHE, triple coupling and doublet peaks corresponding to ^14^NH_4_^+^ and ^15^NH_4_^+^ were detected in the ^1^H NMR spectra of the electrolytes containing ^14^NO_3_^−^ and ^15^NO_3_^−^, respectively (Figure 3g). Cu_2_NCN showed higher Faradaic efficiency (FE) and ammonia yield at each potential than the Cu sample. For the Cu_2_NCN sample, the maximum NH_3_ FE reaches 99.0% at −0.7 V versus RHE and NH_3_ yield is as high as 16 mg h^−1^ cm^−2^ at −0.8 V versus RHE, both of which are significantly higher than that for Cu (Figure 3h,i and Figure S11, Supporting Information).^[^ ^10^ ^]^

The high performance of Cu_2_NCN electrode was well‐sustained during the cycling test (Figure 3j). After the electrolysis, the XRD measurement indicated that most of Cu_2_NCN was preserved, with only a small fraction of metallic state Cu formed. The IR spectra also confirm that asymmetric [N−C≡N]^2−^ anions were retained during the electrolysis (Figure S12,Supporting Information). The production cost per kilo ammonia over Cu_2_NCN is calculated as $ 0.38, which is lower than for other reported electrocatalysts and the commercial price of ammonia produced by the Haber–Bosch process (Figure 3k and Table S3, Supporting Information). Such exceptional performance positions our electrocatalyst as highly competitive when compared to state‐of‐the‐art transition metal‐based electrocatalysts used for NO_3_RR under neutral low‐concentration nitrate solutions (Figure 3l and Table S3, Supporting Information).

### 2.4 NO_3_RR Mechanism for Polarized Cu_2_NCN

**Figure 4:**
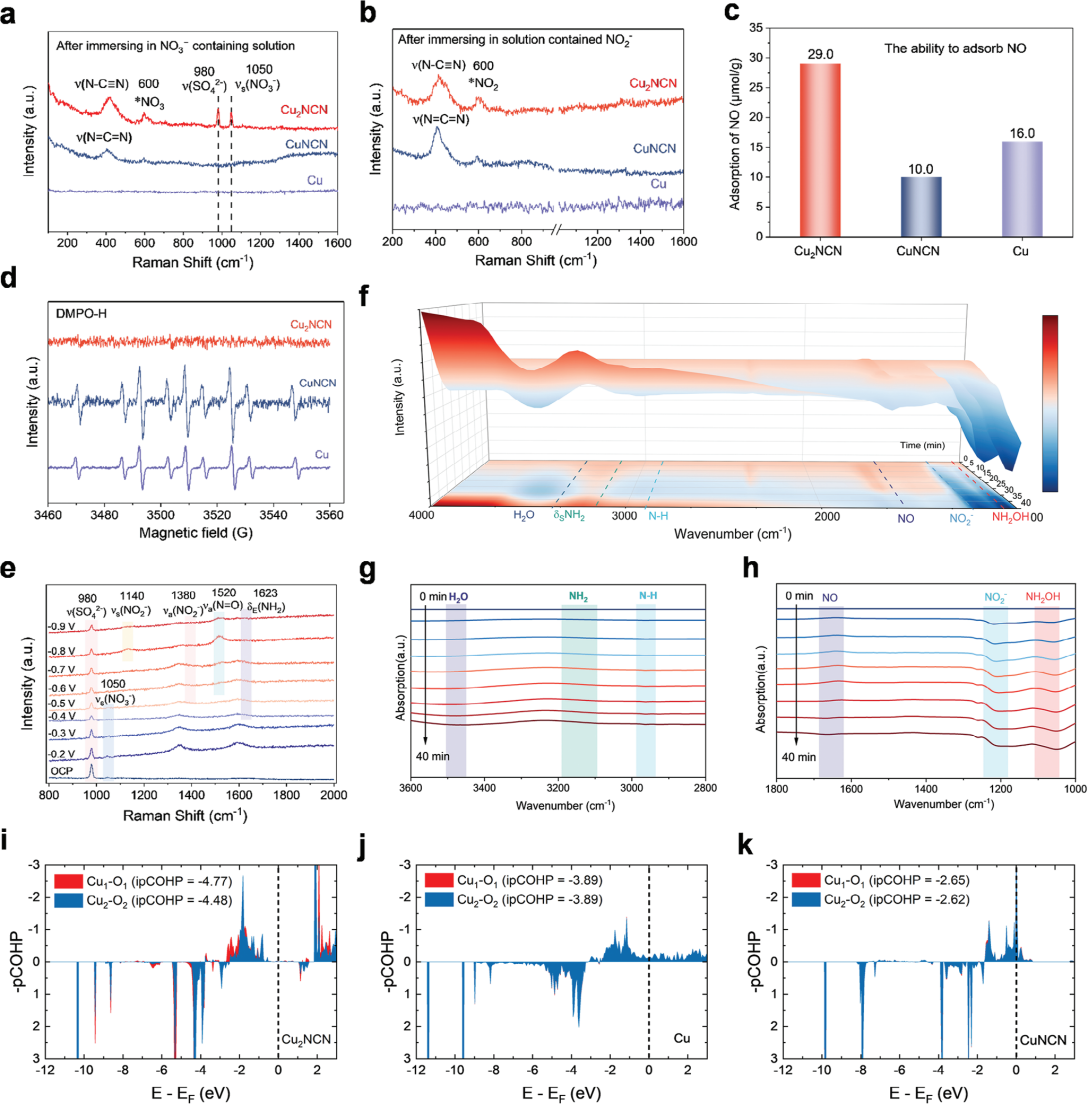
NO_3_RR mechanism studies for polarized Cu_2_NCN. a,b) Raman spectra of Cu, CuNCN, and Cu_2_NCN after immersing in (a) NO_3_^−^ containing electrolyte and (b) NO_2_^−^ containing electrolyte for 24 h. c) NO adsorption of Cu, CuNCN, and Cu_2_NCN determined by TPD measurements. d) ESR spectra for Cu, CuNCN, and Cu_2_NCN at −0.7 V (vs. RHE) after 5 min of NO_3_RR in 0.5 m K_2_SO_4_ with 500 ppm KNO_3_ electrolyte containing DMPO. e) In situ Raman spectra of Cu_2_NCN during NO_3_RR at different potentials in a neutral electrolyte containing 500 ppm KNO_3_. f–h) In situ FT‐IR spectra of Cu_2_NCN under different reaction periods (0–40 min) of NO_3_RR at −0.7 V vs. RHE. i‐k, Crystal Orbital Hamilton population (COHP) analysis for Cu−O coupling characteristics of i) Cu_2_NCN, j) CuNCN, and k) Cu surfaces. The Fermi level is set to zero at the dotted line.

We thoroughly investigated the asymmetric effect of polarized Cu_2_NCN in relation to NO_3_RR. Raman spectra of Cu_2_NCN, CuNCN, and Cu after immersing in NO_3_^−^ or NO_2_^−^ containing electrolytes were collected to investigate the adsorption ability of nitrogen oxides. The peak at 1050 cm^−1^ can be classified as the symmetric NO_3_^–^ stretching associated with adsorbed NO_3_^−^ (**Figures** **4**a and S13, Supporting Information). Similar peak positions were obtained in the concentrated NO_2_^−^ solutions, showing clearer and stronger signals at 600 cm^−1^ of NO_2_^−^ in Cu_2_NCN (Figure 4b and Table S4, Supporting Information). This suggests Cu_2_NCN has enhanced adsorption of NO_3_^−^ and NO_2_^−^ compared to CuNCN and Cu. Moreover, Temperature Programmed Desorption (TPD) was used to investigate the adsorption performance of NO attached to Cu_2_NCN (Figure 4c and Figure S14, Supporting Information). The results showed that Cu_2_NCN exhibits the strongest adsorption capacity of NO in comparison to CuNCN and Cu in line with that the *V*_S_(r) of Cu_2_NCN is most favorable for the adsorption of Lewis bases.

In order to probe whether the H radicals (H^*^) existed during the NO_3_RR process, we monitored the formation of H^*^ upon running NO_3_RR by Electron Paramagnetic Resonance (EPR) using dimethyl‐1‐pyrroline‐N‐oxide (DMPO) as the radical trapping reagent.^[^ ^11^ ^]^ Notably, in the presence of nitrate, the DMPO‐H signals of Cu and CuNCN are still present, while those of Cu_2_NCN are almost undetectable (Figure 4d). This result indicates that the generated H^*^ on the Cu_2_NCN surface participates in the hydrogenation of the activated nitrogen‐containing intermediates on the adjacent surface and is rapidly consumed.

The reaction kinetics was probed by monitoring the evolution of reaction intermediates as a function of reaction time using in situ Fourier transform infrared (FT‐IR) spectroscopy measurement. The in situ FT‐IR spectra were collected during electrolysis at –0.7 V vs. RHE for various time lengths (0–40 min) (Figure 4f,g,h). The strong downward band at 2880 cm^−1^ is attributed to N–H (Figure S15, Supporting Information), in which these signals may come from the intermediates such as –NO_2_H, –NOH, and –NH_2_OH.^[^ ^12^ ^]^ Consequently, Cu_2_NCN might exhibit better proton supply ability during the hydrogenation process than Cu. The in situ electrochemical Raman experiments were also conducted to reveal possible reaction intermediates in the NO_3_RR process (Figure 4e and Figure S16, Supporting Information). The peak signals of the ^*^NHO, ^*^NO_2_ and ^*^NH_2_ quickly increase and decrease, indicating these intermediates were quickly consumed. The iCOHP data matches with the variation in adsorption‐free energy of NO_3_^*^ with structure. More importantly, the interactions of Cu−O_1_ and Cu−O_2_ within NO_3_^*^−Cu (Figure 4j) or NO_3_^*^−CuNCN (Figure 4k) fall into antibonding region across the E_F_, particularly between −1 to 1 eV, accounting for the weaker NO_3_^*^ adsorption on the Cu and CuNCN systems. This binding behavior is quantitively validated by ipCOHP values, which the summed ipCOHP values for Cu−O_1_ and Cu−O_2_ are less negative in the order Cu_2_NCN (−9.25) < Cu (−7.78) < CuNCN (−5.27) (Figure 4i–k), indicating stronger NO_3_^*^ interactions with the Cu_2_NCN catalytic surface. Both the in situ Raman and in situ FT‐IR measurements and computational results consistently suggest that polarized Cu_2_NCN has faster NO_3_RR kinetics than Cu.

### 2.5 Theoretical Calculations on the NO_3_RR Mechanism

**Figure 5:**
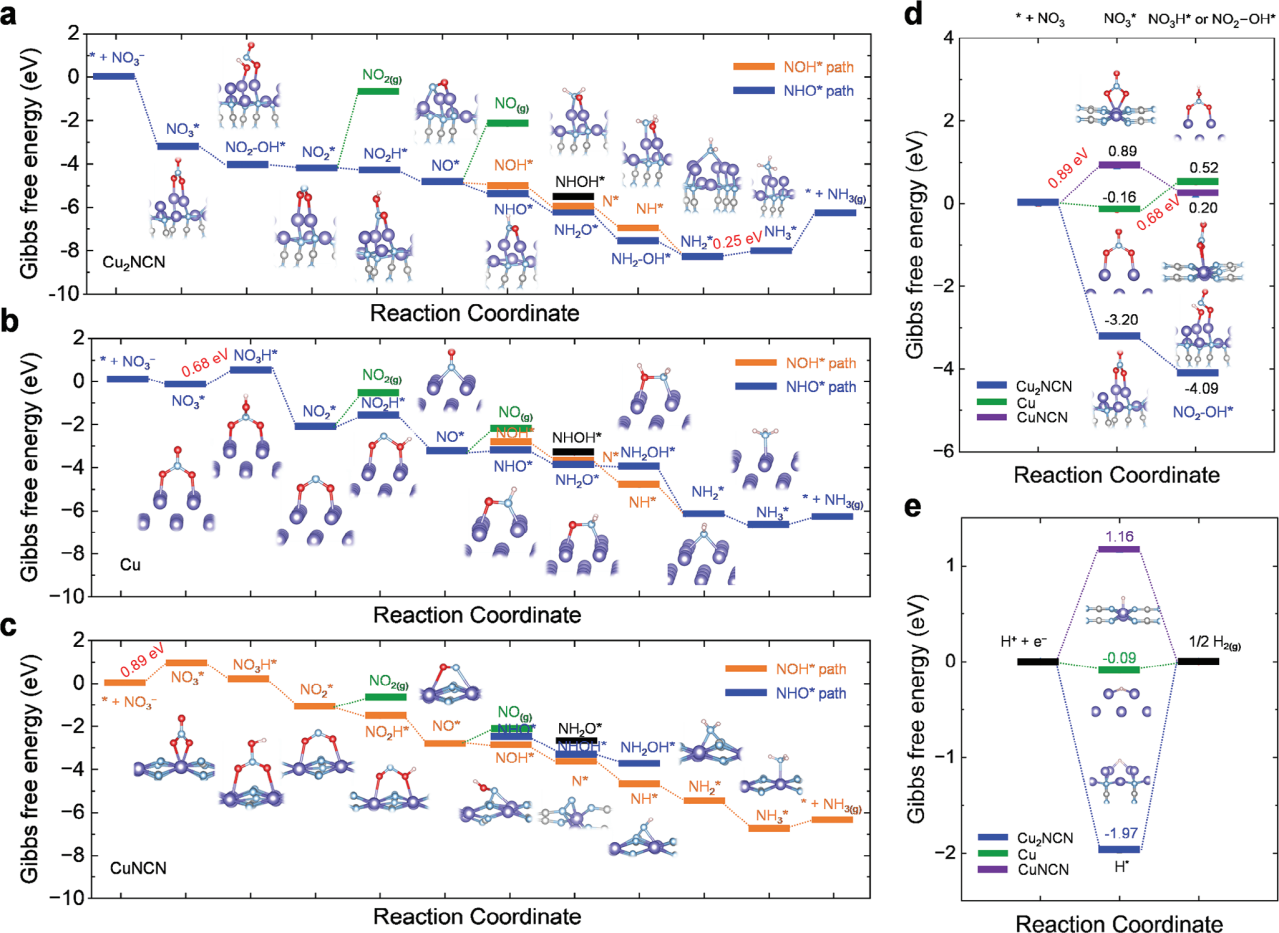
Theoretical calculations on the NO_3_RR mechanism. a–c) Reaction free‐energy profile of NO_3_RR on (a) Cu_2_NCN (100), (b) Cu (100) and (c) CuNCN (100) surface. Optimized configurations of intermediates are shown in the most thermodynamically favorable pathway. Color code: Cu‐purple, N‐light blue, C‐gray, O‐red and H‐pink. d) Calculated Gibbs free‐energy profile for NO_3_^*^ adsorption and subsequent steps for the formation of NO_3_H^*^ on CuNCN(100) and Cu(100), and NO_2_−OH^*^ on Cu_2_NCN(100). e) Calculated Gibbs free‐energies of H^*^ adsorption on Cu_2_NCN(100), CuNCN(100) and Cu(100) to form H_2_ molecules. All insets show the optimized geometries of the intermediates on the Cu sites of Cu, CuNCN, and Cu_2_NCN.

To gain insight into the NO_3_RR mechanism, DFT calculations were conducted to investigate the NO_3_RR activity on Cu_2_NCN(100) (i.e., the strongest peak in the XRD), in comparison with Cu(100) and CuNCN(100).^[^ ^13^ ^]^ The Gibbs free energy diagrams that depict the possible NO_3_RR pathways on Cu_2_NCN(100) clarify the corresponding NO_3_RR mechanisms (**Figure** **5**a and Table S5, Supporting Information). Following the thermodynamically favorable NO_3_^−^ asymmetric adsorption (−3.2 eV) on Cu_2_NCN, the first hydrogenation step of NO_3_^*^ results in the smooth dissociation of the O−N bonds to form NO_2_−OH^*^ with a significant decrease of Gibbs‐free energy (from −3.2 to −4.09 eV) due to the asymmetric configuration of the NO_3_^*^ intermediate (Figure 5a and Figure S17, Supporting Information).

By contrast, the formation of NO_3_H^*^ on Cu is endergonic with a high energy cost of 0.68 eV (Figure 5b), and the NO_3_ adsorption on CuNCN is thermodynamically unfavorable with large energy (0.89 eV) (Figure 5c), thereby serving as the rate‐determining step for the NO_3_RR on Cu(100) and CuNCN(100) (Figure 5d). The accumulated energy for the formation of NO_3_H^*^ intermediate is endergonic on both symmetric Cu and CuNCN (Figures S18,S19, Supporting Information). The nitrate adsorption‐free energies (ΔG_NO3_^*^) of Cu_2_NCN, CuNCN, and Cu (i.e., −3.20, 0.89 and −0.16 eV, respectively) are significantly lower than those of H^*^ adsorption (∆G_H_^*^) (i.e., −1.97, 1.16 and −0.09 eV, respectively), implying a much stronger affinity for NO_3_^−^ over H (Figure 5e). The favorable NO_3_^–^ adsorption on the catalytic surface in the NO_3_RR may potentially block the active site for competitive H^*^ adsorption in the HER, suggesting that NO_3_RR is highly favored over HER.

Partial density of states (PDOS) analysis for O‐2p orbitals of NO_3_^*^ adsorbed species and Cu‐3d orbitals of Cu_2_NCN surfaces also confirm asymmetrical adsorption of NO_3_^*^ and stronger binding compared to nonpolar Cu and CuNCN (Figure S20, Supporting Information). Our computational results strongly support the notion that polarized Cu_2_NCN effectively enhances the hydrogenation process in the NO_3_RR.

### 2.6 Techno‐Economic Analysis of the PER System

**Figure 6:**
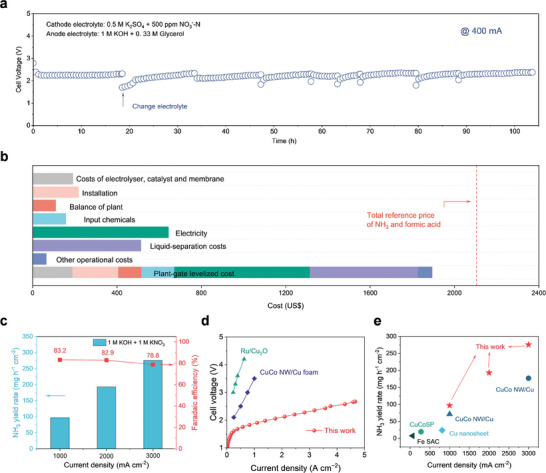
Techno‐economic analysis of PER system. a) Continuous tests of the Cu_2_NCN catalyst for over 100 h using a 4‐cm^2^ MEA‐based electrolyzer for the PER process with neutral electrolyte at the cathode. b) TEA for NH_3_ and formic acid production based on the PER process. Breakdown of the plant‐gate levelized cost per tonne of NH_3_ and the corresponding quantity of formic acid produced on Cu_2_NCN. The TEA findings are calculated based on the NO_3_RR performance for the PER with 4 cm^2^ MEA at 2.4 V. The total reference price of NH_3_ and formic acid is marked by the red dashed line. c) Ammonia yield rate and FE at high current densities in the PER with alkaline electrolyte at the cathode. d) Comparing *V−I* curves of the present PER system at industrial current densities with those in the literatures. e) Comparison of ammonia yield rate at different current densities with previously reported works.

The PER system with Cu_2_NCN as the NO_3_RR cathode and NiCo_2_O_4_ as the GOR anode adopts a neutral electrolyte (0.5 m K_2_SO_4_ with 500 ppm KNO_3_−N) at the cathode side and an alkaline electrolyte (1 m KOH with 0.33 m glycerol) at the anode side for paired electrosynthesis, showing the simultaneous production of value‐added ammonia and formic acid. This PER system could work at a stable full‐cell potential of ≈2.4 V with total currents of 400 mA for over 100 h (**Figures** **6**a and S21, Supporting Information). Under the continuous operation conditions, the NH_3_ FE and formic acid FE are ≈94% and 96%, respectively (Figure S12 and Table S6, Supporting Information).

Our cost analysis encompasses various factors, including the separation of liquid products from one another, expenses associated with the electrolyzer, catalyst, membrane, installation, balance of plant, input chemicals, electricity, as well as other operational costs (e.g., labor and maintenance; see Supporting methods for detailed information).^[^ ^14^ ^]^ It shows that the plant‐gate levelized cost for 1 tonne of NH_3_, plus the corresponding quantity of formic acid produced at 100 mA cm^−2^ on the anodic electrode, is projected to be less than the sum of their reference prices (Figure 6b). The profit is estimated to $211.17 for per tonne of NH_3_ and the corresponding quantity (2.76 tonnes) of formic acid. This result suggests that the PER for continuous electrosynthesis of ammonia and formic acid is profitability, suggesting commercial prospects of the paired valorization route.

Besides, the NH_3_ yield rates and FE under industrial current densities are investigated (Figure 6c). The constructed PER system enables the ampere‐level current density, where the NH_3_ yield rate could reach up to 97000 µg_NH3_ h^−1^ cm^−2,^ and the FE could reach 83.2% under 1000 mA cm^−2^. Moreover, the coupled MEA can maintain 78.8% of FE and the NH_3_ yield rate could reach the astonishing 275.6 mg_NH3_ h^−1^ cm^−2^ at the current density of 3000 mA cm^−2^. As shown in Figure 6d, this PER reactor requests an obviously lower voltage relative to the reported NH_3_ production, achieving 1000 mA cm^−2^ under ≈2V, which is one of the highest ammonia yield rates per unit area reported to date (Figure 6e).

## 3 Conclusion

In summary, our study highlights the exceptional performance of the polarized Cu_2_NCN catalyst in NO_3_RR when coupled with GOR in a PER at industrial‐level currents. Over 100 operational hours, this catalytic system consistently achieves highly efficient production of ammonia and formic acid. Through a strategic combination of in situ electrochemical experiments and theoretical calculations, we elucidate that the exceptional NO_3_RR ability of Cu_2_NCN could be ascribed to asymmetric adsorption, promoting efficient N−O bond cleavage and enhancing ammonia selectivity. Moreover, aiming at industrial NH_3_ production, our paired electro‐refinery MEA exhibits superior 4000 mA cm^−2^ at 2.52 V and remains stable at industrial‐level 1000 mA cm^−2^ for 100 h with the NH_3_ production rate 97000 µg_NH3_ h^−1^ cm^−2^ and a Faradaic efficiency of 83%. And this PER could reach the astonishing 275.6 mg_NH3_ h^−1^ cm^−2^ at the current density of 3000 mA cm^−2^. TEA confirms the economic viability of our PER system for industrial, continuous, and profitable electrosynthesis of green ammonia and valuable anodic products. This research offers a compelling pathway for advancing the decarbonization of the petrochemical industry.

## 4 Experimental Section

### Cu_2_NCN Synthesis

Copper chloride (2.5 mmol) was first dissolved in deionized water (50 mL) at room temperature, followed by adding NaOH (8 mmol) and H_2_NCN (5 mmol). After stirring, N_2_H_4_ (3 mL) was added into the solution dropwise. After 1 h, the product was obtained by vacuum freeze–drying after filtration and washed with deionized water.

### CuNCN Synthesis

Copper chloride (2.5 mmol) was first dissolved in deionized water (50 mL) at room temperature, followed by adding NaOH (8 mmol) and H_2_NCN (5 mmol). Finally, the product was obtained by vacuum freeze–drying after filtration and washed with deionized water.

### Electrochemical Measurements

Electrochemical measurements were performed in a standard three‐electrode system (Jiangsu BOKE New Materials Technology Co., LTD.) using a CHI 760E electrochemical workstation. A Hg/HgCl_2_ electrode and a graphite rod were used as the reference electrode and counter electrode, respectively. The catalyst ink was prepared by dispersing 10 mg catalyst (Cu_2_NCN) and 2 mg Super P carbon black in 1000 µL ethanol containing 50 µL of 5 wt.% Nafion, followed by sonication for 45 min to generate the catalyst ink. The as‐prepared electrodes were used as the working electrode with catalyst loading of 2 mg cm^−2^ on carbon felt. Fumasep FAB‐PK‐130 was used as an anion exchange membrane (AEM), provided by Jiangsu BOKE Co., LTD. All the potentials vs. Hg/HgCl_2_ were converted to the values versus reversible hydrogen electrode (RHE) according to the equation (E vs. RHE = E vs. Hg/HgCl_2_ +0.0592 × pH + 0.244 V). Linear sweep voltammetry (LSV) polarization curves were recorded at a scan rate of 5 mV s^−1^ and 500 rpm. For the catalytic potential, we did not use iR correction. Before the LSV test, we conducted ten cycles of cyclic voltammetry measurements (100 mV s^−1^) to clean the catalyst surface. Unless otherwise stated, all linear voltammetry curves were recorded after three prescans to achieve stabilization.

### Membrane Electrode Assembly (MEA) Tests in a Paired Electro‐Refinery

For testing in a flow electrolyzer (Model: BKT2‐SN‐22‐8X, manufactured by Jiangsu BOKE Co., Ltd., China), we prepared a membrane electrode assembly (MEA) by sandwiching the Cu_2_NCN cathode and NiCo_2_O_4_ anode between a commercial membrane (Fumasep FAB‐PK‐130). The MEA was then placed within a custom‐designed electrolyzer where 500 ppm KNO_3_–N with 0.5 m K_2_SO_4_ /1 m KOH with 1 m KNO_3_ as the catholyte and 1 m KOH with 0.33 m glycerol as the anolyte was circulated through cathode and anode, respectively at a flow rate of 60 mL min^−1^.

### TPD for NO Adsorption

All TPD studies were conducted in a quartz reactor. Typically, 50 mg of adsorbent was loaded into the reactor. A quartz wool plug was placed below the bed to prevent the adsorbent from entering the effluent gas line. Before NO adsorption measurements, the adsorbent underwent hydrothermal aging (HTA) in a stream of air containing 5% water from 348 to 773 K at 2 K min^−1^. The temperature was held at 773 K for 5 h, and then cooled back to 348 K in the absence of water vapor.

### In situ Raman Analyses

In situ Raman spectra were recorded on a micro‐Raman spectrometer (Renishaw) under an excitation of 532 nm laser light under controlled potentials by the CHI 630E electrochemical workstation. The electrochemical operando Raman Cell was provided by the Beijing Scistar Technology Co., Ltd. In addition, Cu_2_NCN deposited on glassy carbon was used as a working electrode. A Pt wire as the counter electrode was rolled to a circle around the cell. Ag/AgCl electrode (sat. KCl) was used as the reference electrode. The in situ Raman spectra were collected under chronoamperometry (I‐t) at different potentials in 0.5 m K_2_SO_4_ with 500 ppm N–KNO_3_ solution.

### In situ IR Analyses

The in situ infrared spectroscopy measurements were carried out on a Nicolet 6700 FT‐IR spectrometer (Thermo Scientific, USA) equipped with a liquid‐nitrogen cooled MCT‐A detector. For continuous collection of in situ FT‐IR spectra during NO_3_RR, the catalyst (10 µg) was supported on a glassy carbon electrode, which served as the working electrode. A saturated Hg/HgO electrode and a Pt wire were used as the reference and counter electrode, respectively. The electrochemical cell was filled with the specific reaction solution. Each spectrum was scanned 32 times with a spectral resolution of 4 cm^−1^ and a time interval of 0.2 min during the electrochemical reduction process.

### XANES and EXAFS Analyses

The Cu K‐edge XAFS were measured at the SXRMB beamline at the Canadian Light Source. The spectra were recorded in fluorescence mode with normalization to the incident photon flux. All spectra were processed using the ATHENA software (version 0.9.26.10).^[^ ^15^ ^]^ EXAFS fitting was performed with FEFF models within an R range of 1 and 2 Å (first shell only). The spectra were obtained through the Fourier transform at k‐range of 3 and 11.7 Å^−1^. The wavelet transformation (W. T.) of χ(k) was conducted on a Python‐based signal.

### Detection of NH_4_^+^

The quantification of NH_4_^+^ was conducted with Nessler's reagent as the coloring agent.^[^ ^16^ ^]^ 1 mL electrolyte after NO_3_RR was first taken out from the cathodic compartment and diluted to 5 mL. Then potassium sodium tartrate solution (500 g L^−1^, 0.1 mL) was added and thoroughly mixed. In the last step 0.1 mL of Nessler's reagent was added to the above mixture. After being left standing for 20 min, the absorbance at 420 nm was measured by UV‐spectroscopy (PG2000‐Pro back‐thinned spectrometer, ideaoptics, China). The obtained value was then fitted to the calibration curve to acquire the corresponding NH_4_^+^ concentration.

### Calculation of NH_3_ Faradaic Efficiency

The NH_3_ Faradaic efficiency was calculated according to

(1) $$FE_{NH3}=\frac{Q_{NH3}}{Q}=\frac{n_{NH3}V_{NH3}F}{Q}$$

where Q represents the applied overall coulomb quantity (C), Q_NH3_ is the coulomb required to produce NH_3_, n is the electron‐transfer number (for 1 mol NH_3_, it is 8), V is the volume of the catholyte of the cathode chamber (30 ml), C_NH3_ is the concentration of NH_3_ produced, and F is the Faraday constant (96,485 C mol^−1^).

### Computational and Simulation Details

Density functional theory (DFT) calculations were implemented using the Vienna ab initio simulation package (VASP)^[^ ^13c^ ^]^ using Perdew, Burke, and Ernzerhof (PBE) functional^[^ ^13b^ ^]^ within generalized gradient approximation (GGA).^[^ ^17^ ^]^ A plane wave cutoff of 500 eV was applied. A 3×3×1 Monkhorst–Pack *k*‐point sampling was used in the first Brillouin zone, whereas the *k*‐point mesh was increased to 5×5×1 for electronic property calculations. A vacuum layer of 15 Å was set to minimize interlayer interactions. The convergence tolerance of energy and force was taken to be 0.02 eV Å^−1^ and 10^−5^ eV, respectively. The DFT‐D3 method of Grimme was used to calculate dispersion corrections.^[^ ^18^ ^]^ The solvation effects were included by using the VASPsol model.^[^ ^19^ ^]^ Bader charge analysis was conducted to calculate the charge population.^[^ ^20^ ^]^ VASPKIT was used for data‐postprocessing of PDOS.^[^ ^13a^ ^]^ The surface electrostatic potential at the 0.001 au electron isodensity contour was computed from CHGCAR and LOCPOT files of VASP. Extended vacuum layers of up to 25 Å were used to minimize the interaction between the slabs and their influence on the surface electrostatic potential, and no dipole correction was used here to properly account for the surface polarization. The electrostatic potential was corrected against the vacuum potential in the middle of the gap. The VESTA program was used for the electrostatic potential plots.^[^ ^21^ ^]^

According to the computational hydrogen electrode (CHE) model,^[^ ^22^ ^]^ the Gibbs free‐energy change (ΔG) was calculated as Δ*G* = Δ*E* + Δ*E*_ZPE_ – *T*Δ*S*, where Δ*E*, Δ*E*_ZPE,_ and Δ*S* are the total energy difference, zero‐point energy change and entropy change (Temperature *T* was set to 298.15 K). To avoid calculating the energy of charged ${\mathrm{NO}}_{3}^{-}$ species, the gas state of HNO_3_ was selected as a reference and the following reactions were considered as:^[^ ^23^ ^]^

(2) $$\mathrm{HN}{O_{3}}_{\left(g\right)}\to \mathrm{HN}{O_{3}}_{\left(\mathrm{aq}\right)}$$

(3) $$\mathrm{HN}{O_{3}}_{\left(\mathrm{aq}\right)}\to {\mathrm{NO}}_{3}^{-}+H^{+}$$

(4) $${\mathrm{NO}}_{3}^{-}+*\to {\mathrm{NO}}_{3}^{*}+e^{-}$$

Consequently, the adsorption‐free energy of ${\mathrm{NO}}_{3}^{-}$ ($\Delta G_{NO_{3}^{*}})$ was described as $\Delta G_{NO_{3}^{*}}=G_{NO_{3}^{*}}+1/2G_{H_{2\;}}-G^{*}-\;G_{HNO_{3(g)\;}}$ following the overall reaction:

(5) $$\mathrm{HN}{O_{3}}_{\left(g\right)}+*\;\to {\;\mathrm{NO}}_{3}^{*}+H^{+}+e^{-}$$

where $G_{NO_{3}^{*}}$, $G_{H_{2\;}}$, G*, and $G_{HNO_{3(g)\;}}$ are the Gibbs free energies of ${\mathrm{NO}}_{3}^{*}$‐adsorbed system, H_2_ gas phase, clean substrate, and HNO_3_ gas phase. Note that the Gibbs free energy change Δ*G_sol_* of Equations (1) and (2) are ‐0.317 and ‐0.075, respectively. Thus, the corrected $\Delta G_{NO_{3}^{*}}$ was then obtained via $\Delta G_{NO_{3}^{*}}^{corr}=\Delta G_{NO_{3}^{*}}+0.392$. Limiting potential (*U*_L_) is defined via the equation: *U*_L_ = −Δ*G*_max_/ne, where Δ*G*_max_ is the free energy difference of the potential determining step and ne is number of electrons transferred in the elementary reaction step.

### Surface Electrostatic Potential Analysis of Polarized Catalyst

Molecular dipoles occur due to the unequal sharing of electrons between atoms in a molecule. When molecules were formed from atoms the valence electrons were polarized toward the more electronegative atoms. The buildup of electron density around an atom or discreet region of a molecule could result in a molecular dipole in which one side of the molecule had a partially negative charge and the other side a partially positive charge. Molecules with dipoles that were not canceled by their molecular geometry are often considered to be polar.^[^ ^24^ ^]^ However, the molecular dipole moment was a very crude estimate of polarity as also molecules that lack a permanent dipole moment interact electrostatically with other molecules.^[^ ^25^ ^]^ In fact even an individual atom of a molecule could be polarized to such an extent that it could interact with both electrophiles (Lewis acids) and nucleophiles (Lewis bases); a phenomenon that was particularly apparent for halogens and was the fundamental reason behind halogen bonding.^[^ ^26^ ^]^ The molecular electrostatic potential (*V*(r)) is physically observable that was much better suited than the molecular dipole moment for analyzing the polarity of molecular systems and their abilities to interact electrostatically with other molecules.^[^ ^25^, ^27^ ^]^ *V*(r) is rigorously defined by

(6)

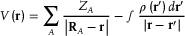

where *Z_A_* is the charge of nucleus A and *r*(r) is the electron density distribution function. *q*V(r) gives the interaction energy between the unperturbed (static) charge distribution of the molecule and a point charge *q* located at the position r. *V*(r) of an atom was everywhere positive and decreases continuously with increasing distance from the nucleus. The electron distribution was rearranged when a molecule was formed from atoms resulting in areas of both positive and negative potential on an isodensity surface surrounding a molecule. Minima in the surface *V*(r), i.e. *V*_S_(r), reflect positions susceptible for attack by electrophiles and maxima in *V*_S_(r) positions for nucleophilic attack.^[^ ^27^ ^]^ The magnitude of *V*_S_(r) at those positions reflects the relative strength of the interactions. Recent studies have also shown that *V*_S_(r) was an effective tool for analyzing the propensity for interactions of crystalline surfaces and nanostructured materials in a manner similar to that for molecular systems.^[^ ^6c^ ^]^ The variability of *V*_S_(r) over the surface was a good indicator of the polarity both for molecular and crystalline systems.^[^ ^6^, ^25^ ^]^

## Conflict of Interest

The authors declare no conflict of interest.

## Author Contributions

J.J.W. and H.T.D.B. contributed equally to this work. J.W., T.B., F.H., and M.Y. proposed and supervised the project. J.J.W. performed material synthesis and the electrochemical experiments. H.T.D.B., T.B. and J.J.W. performed DFT calculations. H.H. and S.K. helped to carry out in situ experiments. X.W., H.Z., and J.M. helped with synthesis of the catalysts and collected the data. J.J.W. and J.X. drew the schematic diagram. Y.L. and L.L. collected the XAFS data and analyzed the data. W.C. and H.B. contributed to data analysis and valuable discussion of the work. J.W. and J.J.W. wrote the manuscript. All authors discussed the results and assisted with manuscript preparation.

## Supporting information



Supporting Information

## Data Availability

The data that support the findings of this study are available on request from the corresponding author. The data are not publicly available due to privacy or ethical restrictions.
