# Supplementary material for: Industrial‐current Ammonia Synthesis by Polarized Cuprous Cyanamide Coupled to Valorization of Glycerol at 4,000 mA cm−2
Source: Adv Mater. 2025 Feb 21;37(14):2418451. doi: 10.1002/adma.202418451 (PMC11983258; doi:10.1002/adma.202418451)
Supplement: Supplementary file 1 — Supporting Information [file ADMA-37-2418451-s001.docx]

Supplementary Information

**Industrial-current Ammonia Synthesis by Polarized Cuprous Cyanamide Coupled to Valorization of Glycerol at 4,000 mA cm^−2^**

Jiacheng (Jayden) Wang^1,2,†^, Huong T. D. Bui^3,†^, Huashuai Hu^4^, Shuyi Kong^5^, Xunlu Wang^4^, Hongbo Zhu^1,2^, Junqing Ma^1,2^, Jintao Xu^1,2^, Yihong Liu^6^, Lijia Liu^6^, Wei Chen^7^, Hui Bi^1^, Minghui Yang^4,^*, Fuqiang Huang^1,5,^*, Tore Brinck^3,^*, and Jiacheng Wang^1,8,9,^*

^1^ The State Key Laboratory of High Performance Ceramics and Superfine Microstructure, Shanghai Institute of Ceramics, Chinese Academy of Sciences, Shanghai 200050, China

^2^ Center of Materials Science and Optoelectronics Engineering, University of Chinese Academy of Sciences, Beijing 100049, China

^3^ Department of Chemistry, CBH, KTH Royal Institute of Technology, SE-100 44 Stockholm, Sweden

^4^ School of Environmental Science and Technology, Dalian University of Technology, Dalian 116024, China

^5^ State Key Laboratory of Metal Matrix Composites, School of Materials Science and Engineering, Shanghai Jiao Tong University, Shanghai 200240, China

^6^ Department of Chemistry, Western University, 1151 Richmond Street, London, ON N6A5B7, Canada

^7^ Department of Materials Design and Innovation, University at Buffalo, The State University of New York, Buffalo, NY 14260, USA

^8^ Zhejiang Provincial Key Laboratory for Island Green Energy and New Materials, Institute of Electrochemistry, School of Materials Science and Engineering, Taizhou University, Taizhou 318000, China

^9^ Key Laboratory of Advanced Energy Materials Chemistry (Ministry of Education), Nankai University, Tianjin 300071, China

⁎ Corresponding authors: [jiacheng.wang@tzc.edu.cn](mailto:jiacheng.wang@tzc.edu.cn) (J. Wang); [tore@kth.se](mailto:tore@kth.se) (T. Brinck); [huangfq@sjtu.edu.cn](mailto:huangfq@sjtu.edu.cn) (F. Huang); [myang@dlut.edu.cn](mailto:myang@dlut.edu.cn) (M. Yang)

^†^These authors contributed equally to this work.

**Table of Contents:**

- Supplementary Methods
- Supplementary Figures 1‒21
- Supplementary Tables 1‒6
- Supplementary Note 1

**Supplementary Methods**

**Technoeconomic analysis**

To ascertain the economic potential of the NH_3_ and formic acid (FA) production (**NO_3_RR coupled with GOR**) in a paired electro-refinery (PER) using renewable electricity, we performed a technoeconomic analysis (TEA) based on the modified model from prior reports ^[1]^. Next is the model we used to calculate the plant-gate levelized cost (unit: US$) for the generation of one tonne of NH_3_ and the corresponding quantity of FA produced in a PER system. In the model, we considered the costs for the electrolyzer, catalyst, membrane, installation, balance of plant, input chemicals, electricity and liquid product separation, as well as other operational costs. Here the cost for the distribution is not considered due to the differences in different regions.

Here, as an example, based on the performance (NH_3_ FE: 94%, formic acid FE: 96%, full-cell potential: 2.4 V) achieved on Cu_2_NCN cathodic catalysts at the operation current density of 100 mA cm^-2^ in the A = 4 cm^2^ MEA electrolyzer, the details for the TEA calculation are listed below.

**1. The cost of the electrolyzer, catalyst, and membrane**

Assuming the production capacity of the plant is 100 tonne of NH_3_ per day, the total current needed is:

$$Total current needed \left[ A \right]=\frac{\frac{{NH}_{3} production \left[ \frac{g}{day} \right]}{molecular {weight}_{{NH}_{3}} \left[ \frac{g}{mol} \right]\times86400 \frac{s}{day}}\times electrons transferred \times Faraday^{'}s Constant}{{NH}_{3} FE\left[ decimal \right]}=\frac{\frac{100\times\frac{{10}^{6}g}{day}}{\frac{17.03g}{mol}\times\frac{86400s}{day}}\times8\times96485\frac{C}{mol}}{0.94}=55807625 A$$

Based on the full cell potential (2.4 V) in the experiments (fig. 3G), we can get the consumed power as follows:

$$Power Consumed \left[ W \right]=Total current needed [A] \times Cell voltage [V] = 55807625 A \times2.4 V = 133938.30 kW$$

From the DOE H2A analysis for central grid electrolysis, the electrolyzer cost for the stack component is $250.25/kW with a reference current density of 175 mA cm^-2^ ^[1a]^. Thus, the total electrolyzer cost is:

$$Total Electrolyzer Cost \left( \$ \right)=Power Consumed \left[ kW \right]\times Electrolyzer Cost \left[ \$kW \right]\times\frac{base current density \left[ \frac{mA}{{cm}^{2}} \right]}{input current density \left[ \frac{mA}{{cm}^{2}} \right]}=133938.30 kW\times250.25 \frac{\$}{kW}\times\frac{175\frac{mA}{{cm}^{2}}}{100\frac{mA}{{cm}^{2}}}=\$58656604.26$$

As the total electrolyzer cost above is the one-time cost for the electrolyzer, we need to convert it to a cost for generating one tonne of NH_3_. We assume the lifetime of the electrolyzer is 20 years with no salvage value at the end of the plant’s lifetime and a plant capacity factor of 0.9 which means the plant produces NH_3_ 328.5 days per year. The electrolyzer cost per tonne of NH_3_ is:

$$Electrolyzer cost \left[ \frac{\$}{tonne {NH}_{3}} \right]=\frac{{CRF}_{electrolyzer}\times Total Electrolyzer Cost [\$]}{Capacity factor \times365 \frac{day}{year}\times production \left[ \frac{tonne {NH}_{3}}{day} \right]}$$

Herein, the capital recovery factor (CRF) is based on a discount rate (denoted i; we use 7% for all the CRF calculations) and the material lifetime.

$${CRF}_{electrolyzer}=\frac{{i(1+i)}^{lifetime}}{{(1+i)}^{lifetime}-1}$$

Hence,

$$Electrolyzer cost \left[ \frac{\$}{tonne {NH}_{3}} \right]=\frac{\frac{0.07\left( 1.07 \right)^{20}}{\left( 1.07 \right)^{20}-1}\times\$58656604.26}{0.9 \times365 \frac{day}{year} \times100 \frac{ton {NH}_{3}}{day}}=\boldsymbol{168.55} \frac{\$}{tonne {NH}_{3}}$$

For the catalyst and membrane cost, we assume that their total one-time cost is 5% of the electrolyzer cost with a lifetime of 5 years. We can then reduce to a cost per tonne of NH_3_ using the same method as above:

$$Catalyst and membrane cost\left[ \frac{\$}{tonne {NH}_{3}} \right]=\frac{{CRF}_{catalyst and membrane} \times Total Electrolyzer Cost \left[ \$ \right]\times5\%}{Capacity factor \times365 \frac{day}{year} \times production \left[ \frac{tonne {NH}_{3}}{day} \right]}=\frac{\frac{0.07\left( 1.07 \right)^{5}}{\left( 1.07 \right)^{5}-1}\times\$58656604.26\times0.05}{0.9 \times365 \frac{day}{year} \times100 \frac{ton {NH}_{3}}{day}}=\boldsymbol{21.78}\frac{\$}{tonne {NH}_{3}}$$

**2. Electricity cost**

By assuming the electricity price is 0.02 $/kWh ^[1b]^, the electricity cost per tonne of NH_3_ is:

$$Electricity cost \left[ \frac{\$}{tonne {NH}_{3}} \right] = \frac{Power Consumed [kW] \times24 hours \times electricity price [\frac{\$}{kWh}]}{{NH}_{3} production \left[ \frac{tonne {NH}_{3}}{day} \right]}= \frac{133938.30 kW \times24 hours \times\frac{0.02 \$}{kWh}}{100 \frac{tonne {NH}_{3}}{day}} =\boldsymbol{642.90} \frac{\$}{tonne {NH}_{3}}$$

**3. Liquid separation cost**

Apart from NH_3_, we also consider generated FA as a liquid byproduct that can be sold along with NH_3_. Due to the liquid crossover, liquid from the cathode outlet and anolyte from the anode side will be collected for separation. We assume the aqueous solution will be recirculated until the total volume concentration of NH_3_ and FA reaches 10%. The cost for the liquid separation is calculated using a distillation model ^[1a]^. To simplify the calculation, here we use a higher cost of distillation for the calculation of all liquid product separation cost and will give a more conservative estimate. For NH_3_ and FA, the distillation model uses a reference cost of $4700000 for a flowrate capacity of 1000 L min^-1^ with a scaling factor of 0.7 and a distillation operating cost of $18000.0 per day ^[1a]^.

At 100 mA cm^-2^, the FA FE is 96%. With 100 tonne of NH_3_ produced per day (FE NH_3_= 94%) via NO_3_RR, the quantity of formate produced per day is calculated according to:

$$\frac{\frac{{NH}_{3} production\left[ \frac{g}{day} \right]}{molecular {weight}_{{NH}_{3}}\left[ \frac{g}{mol} \right]}\times electrons transferred}{\frac{FA production \left[ \frac{g}{day} \right]}{molecular {weight}_{FA}}\times electrons transferred}=\frac{{NH}_{3} FE}{FA FE}$$

We find the FA production per day is **276.0** tonne. We can get the flowrate of NH_3_ and FA according to:

$$Product flowrate \left[ \frac{L}{min} \right]=\frac{Production rate \left[ \frac{kg product}{day} \right]\times1000 \frac{L}{m^{3}}}{Product density \left[ \frac{kg}{m^{3}} \right]\times24 \frac{hour}{day}\times60 \frac{min}{hour}}$$

The flowrates of NH_3_ and FA are 112.55 and 157.10 L min^-1^, respectively. The flowrate of aqueous solution for separation once a product concentration of 10% is achieved is:

$$Aqueous solution flowrate \left[ \frac{L}{min} \right]=\frac{Total product flowrate \left[ \frac{L}{min} \right]}{Product concentration [decimal]}=\frac{(112.55 + 157.10)\frac{L}{min}}{0.1}=2696.5 \frac{L}{min}$$

The distillation capital cost is calculated by scaling the reference cost to the flowrate of aqueous solution.

$$Distillation capital cost \left[ \$ \right]=\$4700000 \times\left( \frac{Aqueous solution flowrate \left[ \frac{L}{min} \right]}{1000 \left[ \frac{L}{min} \right]} \right)^{0.7}=\$4700000 \times\left( \frac{2696.5 \frac{L}{min}}{1000 \frac{L}{min}} \right)^{0.7}=\$9411485.01$$

The distillation capital cost per tonne of NH_3_ and the corresponding quantity of FA (denoted $Distillation capital cost \left[ \frac{\$}{tonne {NH}_{3}} \right]$) is written by assuming the distillation facility lifetime is the same as the electrolyzer lifetime:

$$Distillation capital cost \left[ \frac{\$}{tonne {NH}_{3}} \right]=\frac{{CRF}_{electrolyzer} \times Distillation capital cost [\$]}{Capacity factor \times365 \frac{day}{year} \times production \left[ \frac{tonne {NH}_{3}}{day} \right]}=\frac{\frac{0.07{(1.07)}^{20}}{{(1.07)}^{20}-1} \times\$9411485.01}{0.9 \times365 \frac{day}{year} \times100 \frac{tonne {NH}_{3}}{day}}=\boldsymbol{27.04} \frac{\$}{tonne {NH}_{3}}$$

The distillation operational cost per tonne of NH_3_ and the corresponding quantity of FA (denoted $Distillation operational cost \left[ \frac{\$}{tonne {NH}_{3}} \right]$) is:

$$Distillation operational cost \left[ \frac{\$}{tonne {NH}_{3}} \right]=\frac{Aqueous solution flow rate \left[ \frac{L}{min} \right]}{1000 \frac{L}{min} \times production \left[ \frac{tonne {NH}_{3}}{day} \right]} \times18000.0 \frac{\$}{day}=\frac{2696.5 \frac{L}{min}}{1000 \frac{L}{min} \times100 \frac{tonne {NH}_{3}}{day}}\times18000.0 \frac{\$}{day}=\boldsymbol{485.37} \frac{\$}{tonne {NH}_{3}}$$

The liquid separation cost per tonne of NH_3_ and the corresponding quantity of FA (denoted $Distillation seperation cost \left[ \frac{\$}{tonne {NH}_{3}} \right]$) is:

$$Liquid seperation cost \left[ tonne \frac{\$}{{NH}_{3}} \right]=Distillation capital cost \left[ \frac{\$}{tonne {NH}_{3}} \right] + Distillation operational cost \left[ \frac{\$}{tonne {NH}_{3}} \right]=\boldsymbol{27.04} \frac{\$}{tonne {NH}_{3}} + \boldsymbol{485.37} \frac{\$}{tonne {NH}_{3}} = \boldsymbol{512.41}\frac{\$}{tonne {NH}_{3}}$$

**4. The total** **capital costs**

By summing the cost for the electrolyzer, catalyst, membrane, and distillation capital cost, we can get the total capital costs of $217.37 per tonne of NH_3_ and the corresponding quantity of FA (denoted $Total capital cost \left[ \frac{\$}{tonne {NH}_{3}} \right]$).

**5. Installation cost**

Based on the total capital costs, we assume a Lang factor of 1 for the calculation of equipment installation cost. The installation cost per tonne of NH_3_ and the corresponding quantity of FA (denoted $Installation cost \left[ \frac{\$}{tonne {NH}_{3}} \right]$) is:

$$Installation cost \left[ \frac{\$}{tonne {NH}_{3}} \right]=Lang Factor \times Total capital costs \left[ \frac{\$}{tonne {NH}_{3}} \right]=\boldsymbol{217.37}\frac{\$}{tonne {NH}_{3}}$$

**6. Balance of plant (BoP)**

We assume the balance of plant is 50% of the total capital costs. The balance of plant per tonne of NH_3_ and the corresponding quantity of FA (denoted $BoP \left[ \frac{\$}{tonne {NH}_{3}} \right]$) is:

$$BoP \left[ \frac{\$}{tonne {NH}_{3}} \right]= BoP Factor \times Total capital costs \left[ \frac{\$}{tonne {NH}_{3}} \right]= 50\% \times217.37 \frac{\$}{tonne {NH}_{3}} = \mathbf{108.69} \frac{\$}{tonne {NH}_{3}}$$

**7. Input chemicals cost**

For the input chemicals cost, we account for the cost from the consumed H_2_O , and electrolyte. The water price is estimated as $0.8 per tonne based on the 2023 water rates (5 Yuan m^-3^) for the city of Shanghai, China ^[2]^. The cost of water consumed for **NO_3_RR coupled with GOR** per day can be calculated according to:

$${{NO}_{3}}^{-}+{{C_{3}H}_{8}O}_{3}+H_{2}O \to3 HCOOH+{NH}_{3}+{OH}^{-}$$

$$Cost of consumed H_{2}O=\frac{100 tonne {NH}_{3} \times molecular {weight}_{H_{2}O} \left[ \frac{kg}{mol} \right]}{molecular {weight}_{{NH}_{3}} \left[ \frac{kg}{mol} \right]} \times0.8 \frac{\$}{tonne {NH}_{3}}=\frac{100 \times0.018 \frac{kg}{mol}}{0.017\frac{kg}{mol}} \times0.8 \frac{\$}{tonne {NH}_{3}}=\$84.71$$

The cost of consumed water per tonne of NH_3_ and the corresponding quantity of FA (denoted $Cost of consumed H_{2}O \left[ \frac{\$}{tonne {NH}_{3}} \right]$) is:

$$Cost of consumed H_{2}O \left[ \frac{\$}{tonne {NH}_{3}} \right]= \frac{\$84.71}{100 tonne {NH}_{3}}=\boldsymbol{0.84} \frac{\$}{tonne {NH}_{3}}$$

The NO_3_RR reaction electrolyte is 0.5 M K_2_SO_4_ aqueous solution. And the GOR reaction electrolyte is 1 M KOH aqueous solution. We estimate a fixed volume ratio of 120 L electrolyte per m^2^ of electrolyzer based on our lab-scale experiments. The total volume of electrolyte needed is:

$$Volume of electrolyte \left[ L \right]=\frac{Total current needed [mA]}{Current density \left[ \frac{mA}{{cm}^{2}} \right] \times\left( \frac{100cm}{1m} \right)^{2}} \times120 \frac{L}{m^{2}} =\frac{5580762500 mA}{100 \frac{mA}{{cm}^{2}} \times\left( \frac{100cm}{1m} \right)^{2}} \times120 \frac{L}{m^{2}} = 669691.5 L$$

Assuming a price of $493 per tonne for K_2_SO_4_ ^[3]^ and a price of $800 per tonne for KOH ^[3]^, we can get the total cost of electrolyte including the cost of K_2_SO_4_, KOH, and the cost of water according to:

$$Cost of electrolyte \left[ \$ \right]= \frac{Volume of electrolyte \left[ L \right]}{2}\times molecular {weight}_{KOH} \left[ \frac{kg}{mol} \right] \times1 \frac{mol}{L} \times price of KOH \left[ \frac{\$}{kg} \right] +\frac{Volume of electrolyte \left[ L \right]}{2}\times molecular {weight}_{K_{2}{SO}_{4}} \left[ \frac{kg}{mol} \right] \times0.5 \frac{mol}{L} \times price of K_{2}{SO}_{4} \left[ \frac{\$}{kg} \right] + Volume of electrolyte \left[ L \right] \times water price \left[ \frac{\$}{kg} \right] =\frac{669691.5 L}{2} \times0.056 \frac{kg}{mol} \times1 \frac{mol}{L} \times0.8 \frac{\$}{kg} + \frac{669691.5 L}{2} \times0.174 \frac{kg}{mol} \times0.5 \frac{mol}{L} \times0.493 \frac{\$}{kg} + 669691.5 L \times1 \frac{kg}{L} \times0.0008 \frac{\$}{kg} = \$29898.71$$

By assuming an electrolyte lifetime of one year, we calculate a new CRF:

$${CRF}_{electrolyte} = \frac{0.07{(1.07)}^{1}}{{1.07}^{1}-1} = 1.07$$

For producing 1 tonne of NH_3_ and the corresponding quantity of FA, the cost of electrolyte (denoted$Cost of electrolyte \left[ \frac{\$}{tonne {NH}_{3}} \right]$) is:

$$Cost of electrolyte \left[ \frac{\$}{tonne {NH}_{3}} \right] =\frac{{CRF}_{electrolyte} \times Cost of electrolyte [\$]}{Capacity factor \times365 \frac{day}{year} \times production \left[ \frac{{tonne NH}_{3}}{day} \right]} =\frac{1.07 \times\$29898.71}{0.9 \times365 \frac{day}{year} \times100 \frac{tonne {NH}_{3}}{day}} = \boldsymbol{0.97} \frac{\$}{tonne {NH}_{3}}$$

By assuming glycerol price of $110 per tonne ^[2]^, the cost of glycerol for producing 1 tonne of NH_3_ and the corresponding quantity of FA (denoted $Cost of Glycerol \left[ \frac{\$}{tonne {NH}_{3}} \right]$) is:

$$Cost of Glycerol \left[ \frac{\$}{tonne {NH}_{3}} \right] = molar ratio \left[ \frac{Glycerol}{FA} \right] \times\frac{FA \left[ \frac{g}{day} \right]}{60.055 \frac{g}{mol}} \times\frac{92 \frac{g}{mol}}{100\frac{tonne {NH}_{3}}{day}} \times price of Glycerol \left[ \frac{\$}{tonne} \right] = \frac{1}{3} \times\frac{276000000 \frac{g}{day}}{60.055 \frac{g}{mol}} \times\frac{92 \frac{g}{mol}}{\frac{1000000 g}{tonne} \times100 \frac{tonne {NH}_{3}}{day}} \times110 \frac{\$}{tonne} = \boldsymbol{155.03} \frac{\$}{tonne {NH}_{3}}$$

We can get the input chemicals cost per tonne of NH_3_ and the corresponding quantity of FA (denoted $Input chemicals cost \left[ \frac{\$}{tonne {NH}_{3}} \right]$) according to:

$$Input chemicals cost \left[ \frac{\$}{tonne {NH}_{3}} \right] = Cost of consumed H_{2}O \left[ \frac{\$}{tonne {NH}_{3}} \right] + Cost of electrolyte \left[ \frac{\$}{tonne {NH}_{3}} \right] + Cost of Glycerol \left[ \frac{\$}{tonne {NH}_{3}} \right] = \boldsymbol{0.84} \frac{\$}{tonne {NH}_{3}} + \boldsymbol{0.97} \frac{\$}{tonne {NH}_{3}} + \boldsymbol{155.03}\frac{\$}{tonne {NH}_{3}}= \boldsymbol{156.84} \frac{\$}{tonne {NH}_{3}}$$

**8. Other operational costs**

Other operational costs (such as labor and maintenance; denoted $Other operational costs \left[ \frac{\$}{tonne{NH}_{3}} \right]$) are assumed to be 10% of the electricity cost per tonne of NH_3_ and the corresponding quantity of FA:

$$Other operational costs \left[ \frac{\$}{tonne {NH}_{3}} \right] = Electricity cost\left[ \frac{\$}{tonne {NH}_{3}} \right] \times0.1 = 642.90 \frac{\$}{tonne {NH}_{3}} \times0.1 = \boldsymbol{64.29} \frac{\$}{tonne {NH}_{3}}$$

9. **The plant-gate levelized cost**

The plant-gate levelized cost for producing 1 tonne of NH_3_ and the corresponding quantity of FA (denoted $Plant- gate levelized cost \left[ \frac{\$}{tonne {NH}_{3}} \right]$) is:

$$Plant - gate levelized cost \left[ \frac{\$}{tonne {NH}_{3}} \right] =\left( 168.55+21.78+642.90+512.41+217.37+108.69+156.84+64.29 \right) \frac{\$}{tonne {NH}_{3}} = \boldsymbol{1892.83} \frac{\$}{tonne {NH}_{3}}$$

**10. Potential profit**

We assume the reference prices of NH_3_ and FA per tonne are $1000, and $400, respectively ^[2, 4]^. The profit per tonne of NH_3_ and the corresponding quantity of FA is calculated according to:

$$1 tonne \times1000 \frac{\$}{tonne} + \frac{276}{100} tonne \times400 \frac{\$}{tonne} - \$1892.83=\$\boldsymbol{211.17}$$

**Supplementary Figures**


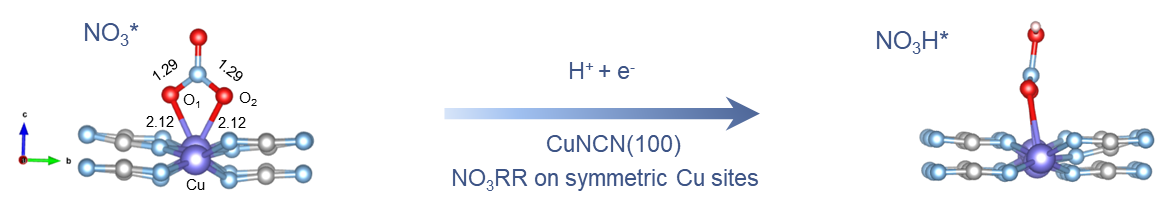


**Supplementary Fig. S1 |** The symmetric adsorption of NO_3_* on CuNCN facilitates the formation of NO_3_H* over hydrogenation with increased difficulty in the O−N bond breaking.


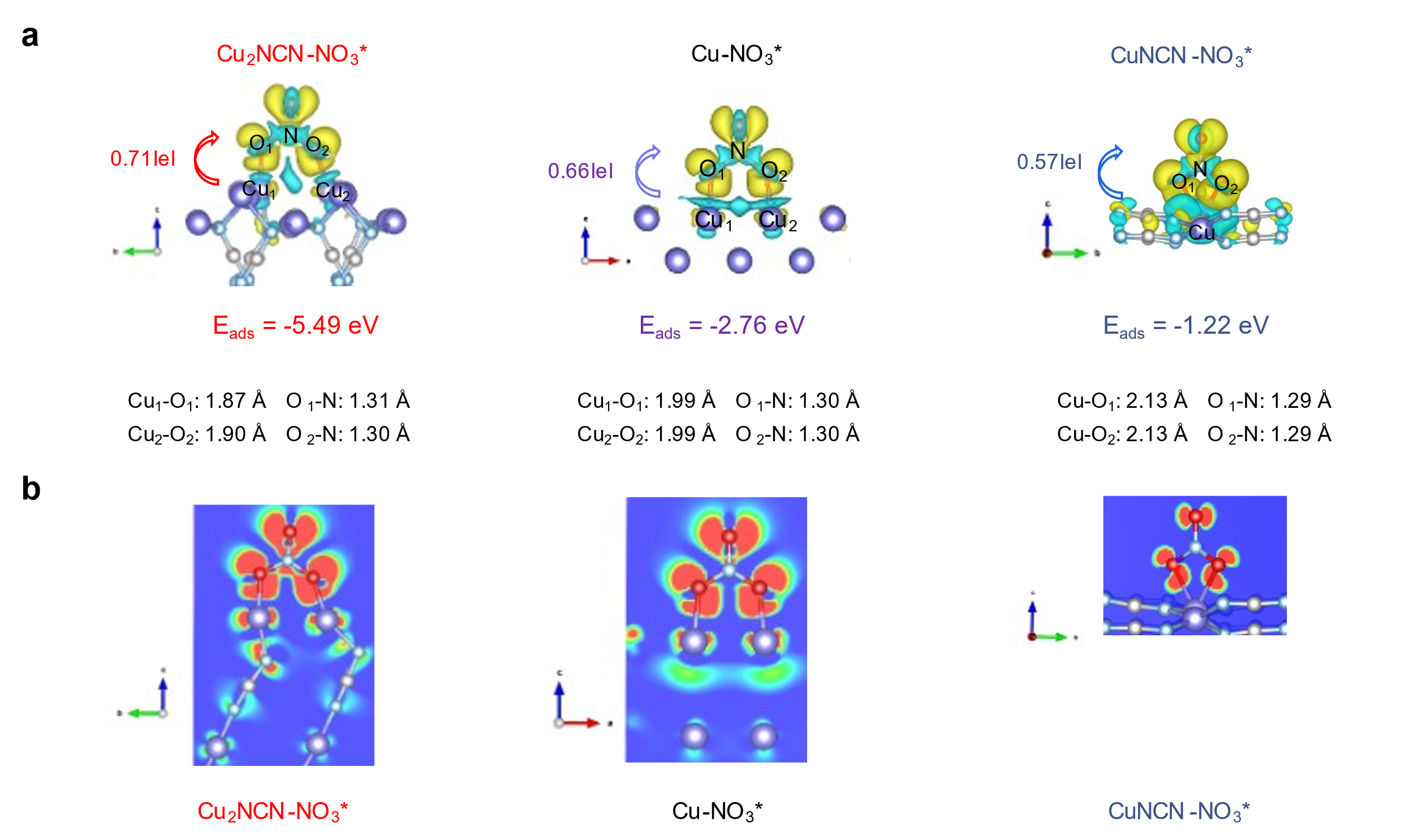


**Supplementary Fig. S2 | Charge density difference of NO_3_* adsorption. (a)** Charge density difference, Cu−O bond length (Å), and adsorption energy for NO_3_* asymmetric adsorption on Cu_2_NCN(100) and NO_3_* symmetric adsorption on Cu(100) and CuNCN(100). On the Cu sites of Cu_2_NCN(100), the NO_3_* asymmetric adsorption is the strongest, which leads to different Cu−O bonds lengths (1.87 Å vs. 1.90 Å) and a slight elongation of the O−N bonds (1.31 Å vs. 1.30 Å). Electron accumulation and depletion are denoted in yellow and cyan, respectively. **(b)** The EDD of NO_3_^–^ on Cu_2_NCN(100) (left), Cu(100) (centre) and CuNCN(100) (right). Purple, blue, gray and red spheres represent the Cu, N, C and O atoms, respectively. The blue electronic cloud indicates charge accumulation and the red electronic cloud indicates charge depletion. A large electron cloud existing between the NO_3_^−^ and Cu_2_NCN(100) indicates a stronger interaction than that on the Cu(100) and CuNCN(100).

**
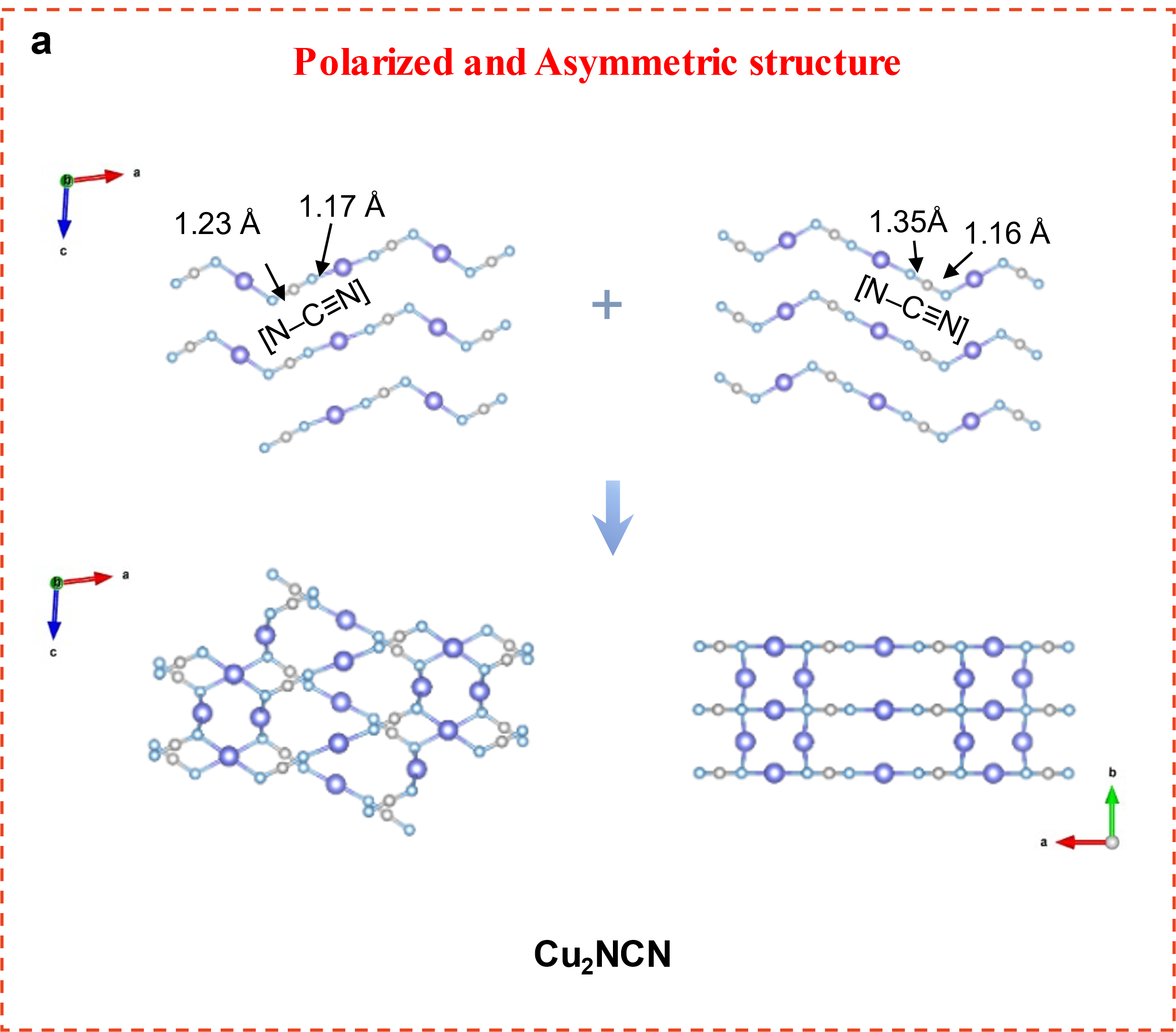
**

**
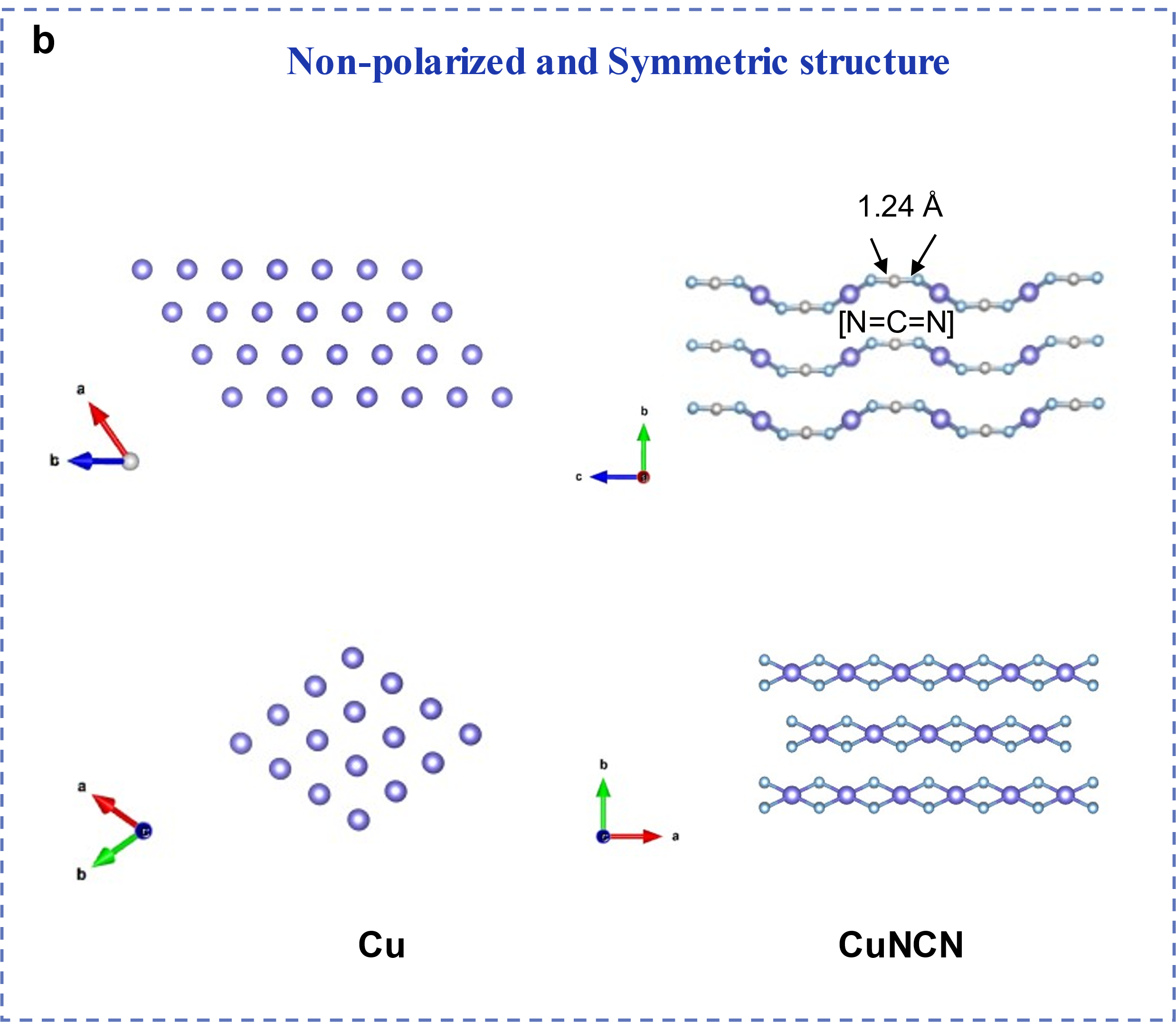
**

**Supplementary Fig. S3** **| Schematic illustration of (a) the polarized Cu_2_NCN, (b) the nonpolarized Cu and CuNCN structure.** Polar Cu_2_NCN (Layer-by-layer stacking, held together by Cu atoms) exhibits an asymmetric structure linked by the asymmetric [N–C≡N]^2−^. Nonpolar Cu and CuNCN linked by [N=C=N]^2−^ exhibit symmetrical structures.

**
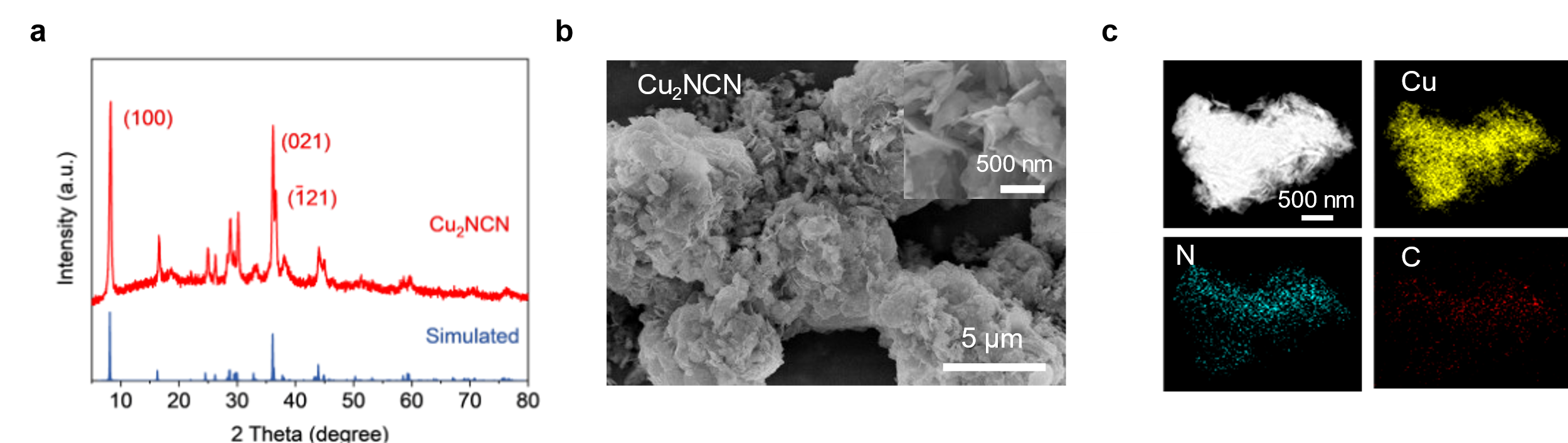
**

**Supplementary Fig. S4 | Structure characterization of Cu_2_NCN.** (**a)** XRD pattern of Cu_2_NCN. (**b)** SEM image of Cu_2_NCN. Cu_2_NCN exhibits a micro-flower morphology composed of nanosheets. (**c)** TEM image and EDX mapping images of Cu_2_NCN. The EDX mapping images show the same distribution of Cu, N, and C elements in Cu_2_NCN.

**
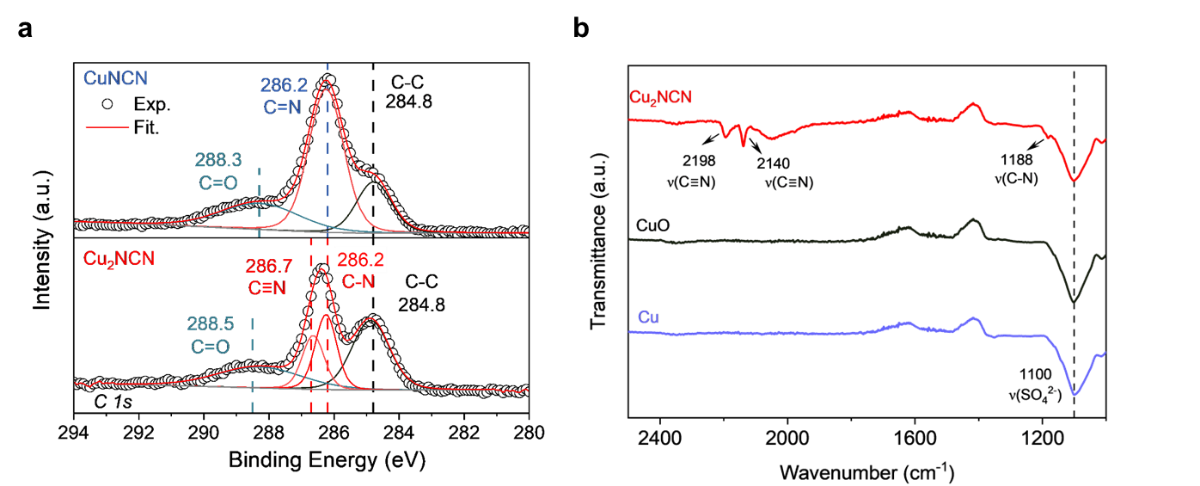
**

**Supplementary Fig. S5 |** **(a)** High-resolution X-ray photoelectron spectroscopy (XPS) of Cu_2_NCN and CuNCN. Deconvoluted spectra of C 1s. Compared to CuNCN with only C=N bonds, Cu_2_NCN exhibits two forms of bond binding (C–N and C≡N). **(b)** Infrared spectra of Cu_2_NCN. IR spectrum of Cu_2_NCN (red curve), CuO (black curve) and Cu (blue curve). Vibration peaks confirm the major type of NCN^2−^ as [N–C≡N]^2−^ in Cu_2_NCN.


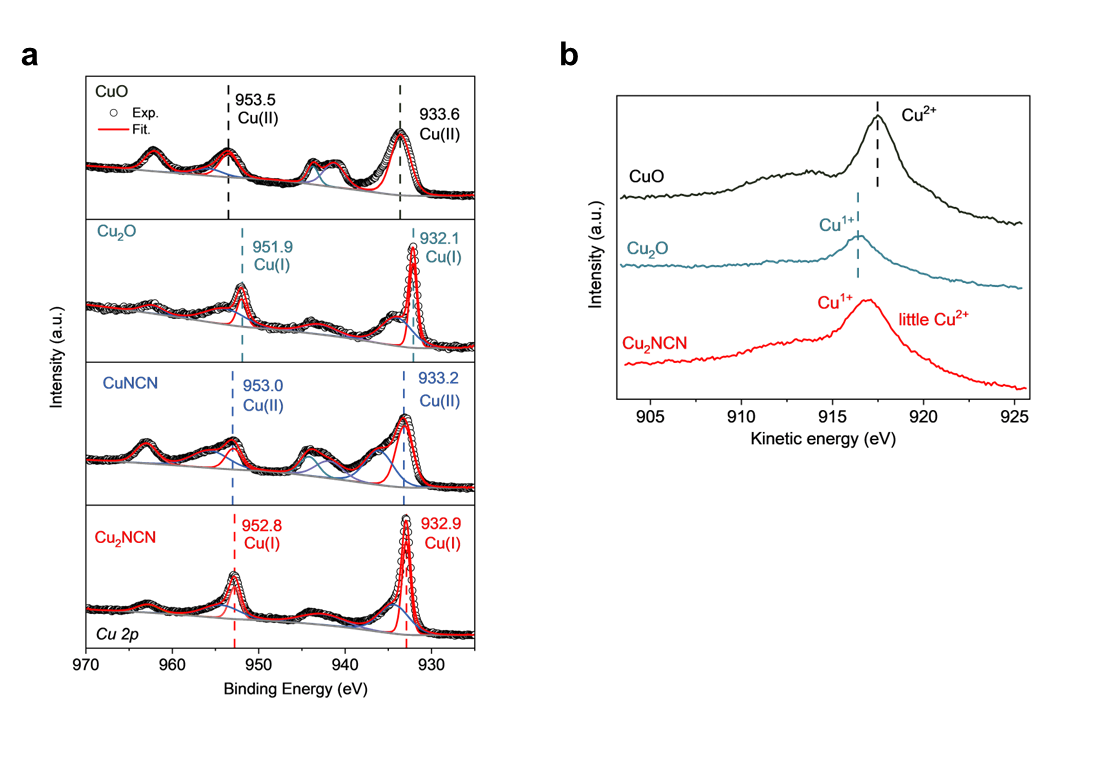


**Supplementary Fig. S6 | (a)** High-resolution X-ray photoelectron spectroscopy (XPS) of Cu_2_NCN, CuNCN, Cu_2_O and CuO. Deconvoluted spectra of Cu 2p. **(b)** Auger electron spectra of Cu_2_NCN Cu_2_O and CuO (differential spectrum). The peak at 916.5 eV indicated the major Cu(I) phase on the Cu_2_NCN surface.


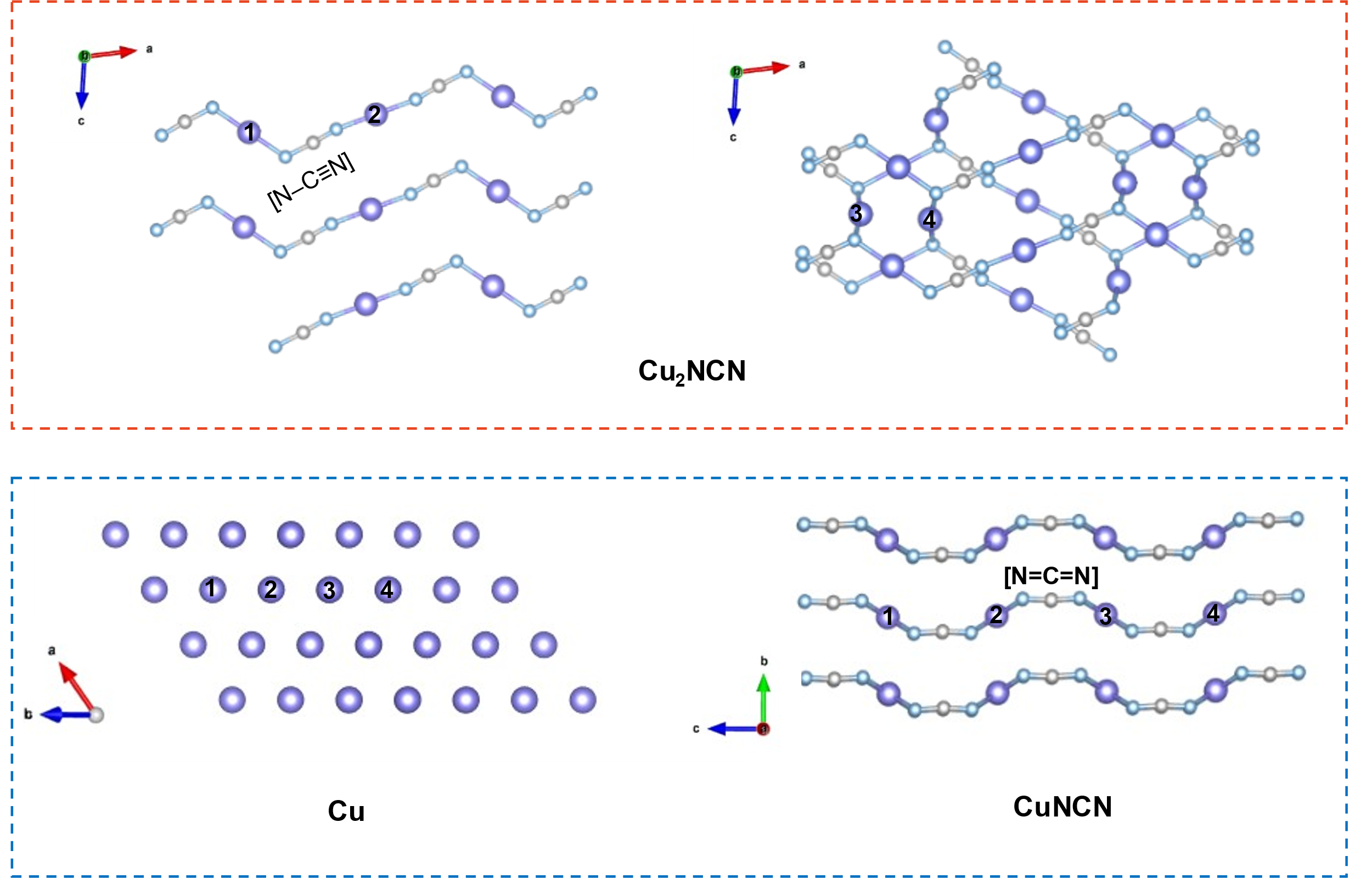


**Supplementary Fig. S7 |** Cu 1, 2, 3, 4 sites represent different positions in the crystal structures of Cu, CuNCN and Cu_2_NCN. The Bader charge distribution confirms the asymmetrical Cu sites. Compared to Cu and CuNCN, Cu2NCN displays different valence states at Cu1 and Cu2 sites.


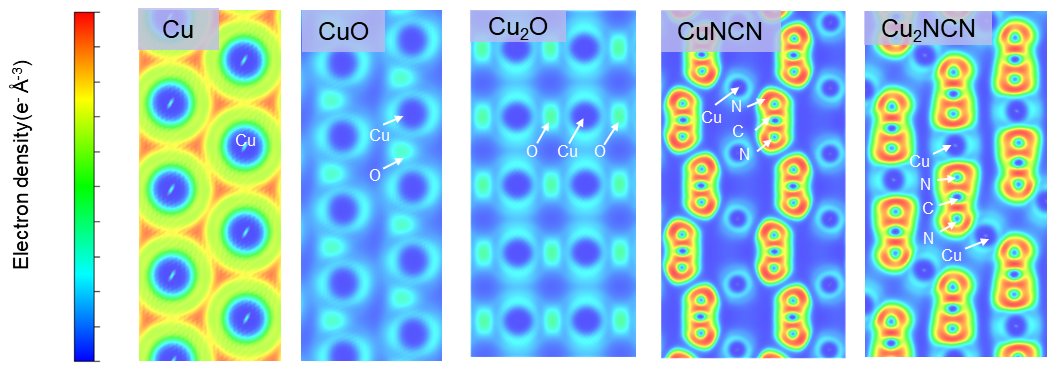


**Supplementary Fig. S8 | Electron localization function patterns of Cu, CuO, Cu_2_O, CuNCN and Cu_2_NCN.** Cu_2_NCN clearly shows asymmetrical electron density states around Cu atoms in comparison to other symmetric Cu-based catalysts.

**
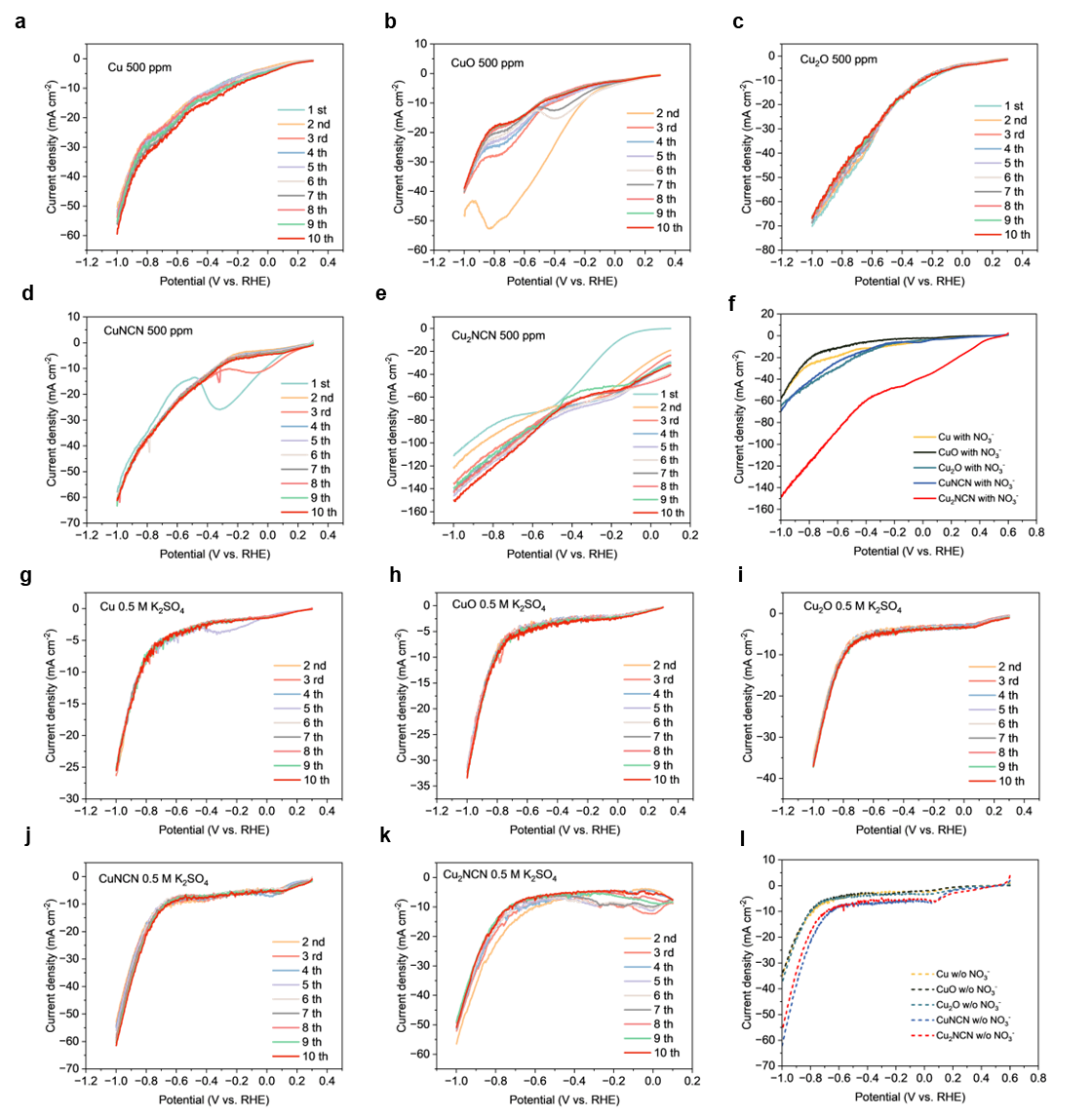
**

**Supplementary Fig. S9 |** The linear sweep voltammetry (LSV) tests. The first 10 LSV for Cu, CuO, Cu_2_O, CuNCN and Cu_2_NCN in 0.5 M K_2_SO_4_ with 500 ppm N–KNO_3_ **(a, b, c, d, and e)** and 0.5 M K_2_SO_4_ **(g, h, i, j, k)**, respectively. LSV curves of the Cu_2_NCN, CuNCN, Cu_2_O, CuO and Cu in **(f)** 500 ppm N–KNO_3_/0.5 M K_2_SO_4_ mixed electrolyte and **(l)** 0.5 M K_2_SO_4_ electrolyte.


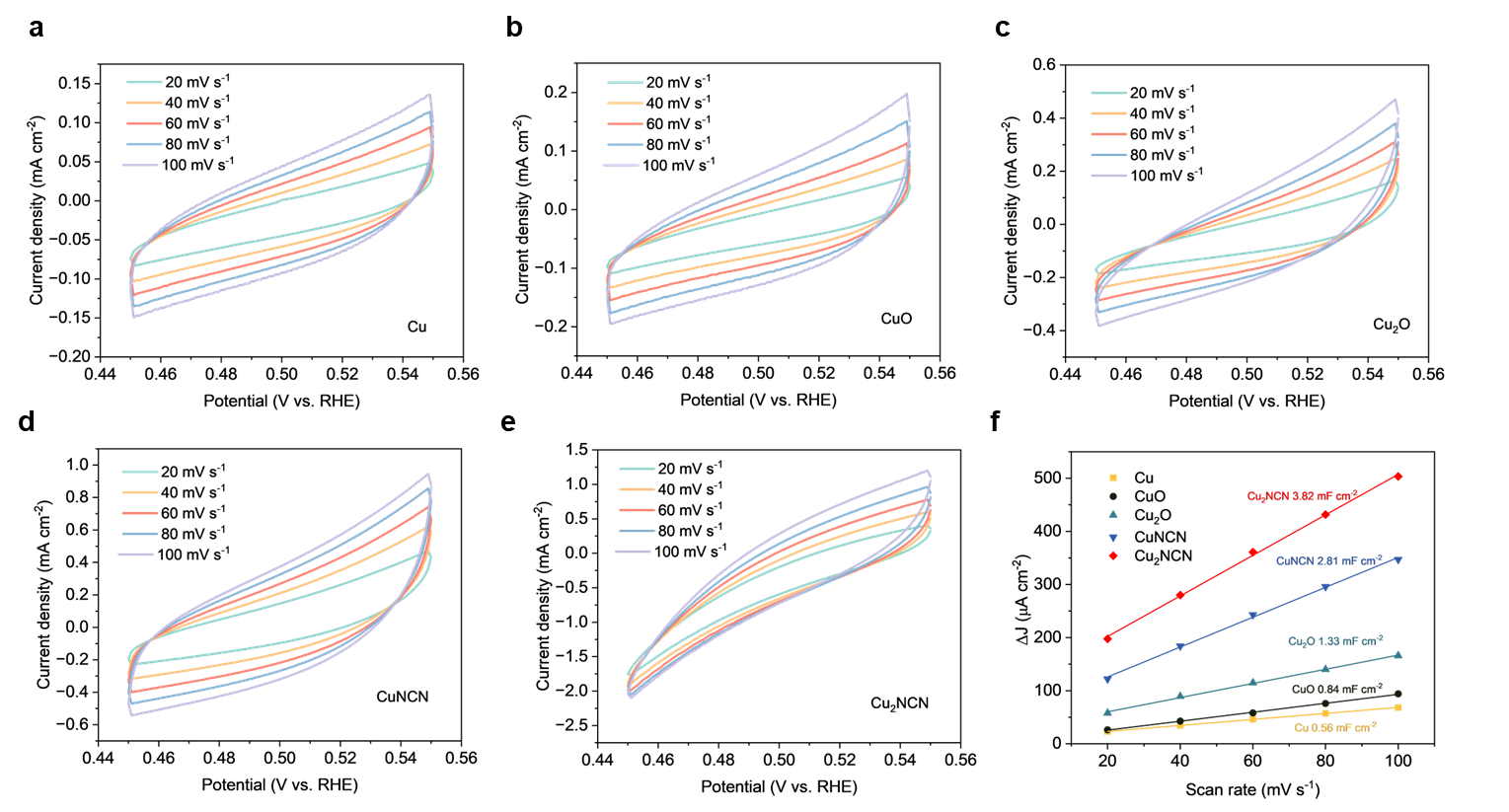


**Supplementary Fig. S10 | ECSA measurements.** CV curves of Cu (a), CuO (b), Cu_2_O (c), CuNCN (d), and Cu_2_NCN (e) with different scan rates of 20, 40, 60, 80, and 100 mV s^−1^.

**
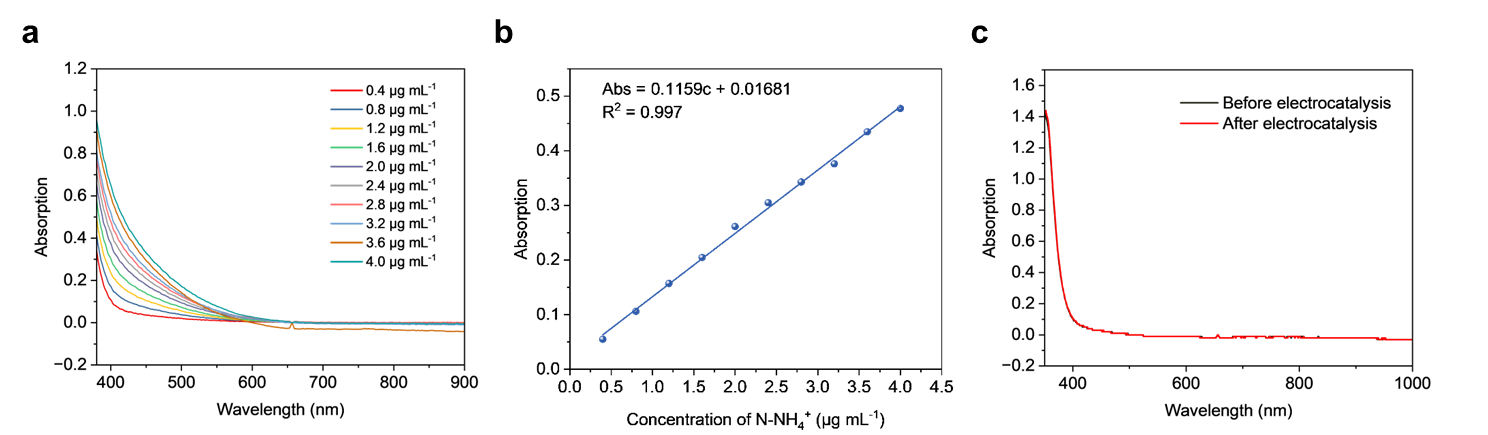
**

**Supplementary Fig. S11 | (a)** UV-vis calibration curve of NH_3_ in ultrapure water using (NH_4_)_2_SO_4_ solutions of known concentration as standards. **(b)** Calibration curve used for estimation of NH_3_. **(c)** UV-vis curves of electrolytes before and after electrocatalysis test for Cu_2_NCN in 0.5 M K_2_SO_4_ solution without KNO_3_.


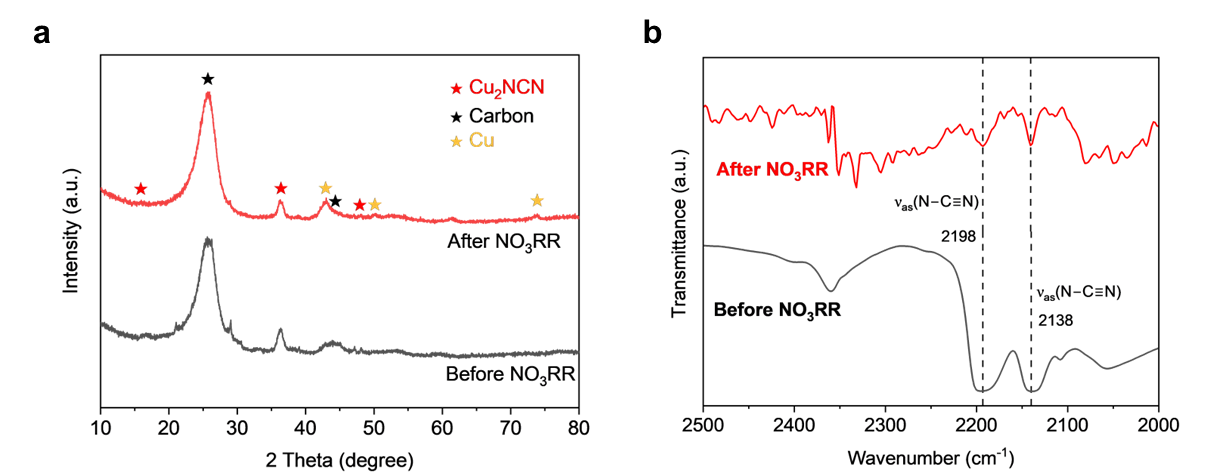


**Supplementary Fig. S12 |** Structure and Spectroscopic characterization on the stability of Cu_2_NCN after the NO_3_RR electrolysis. **(a)** XRD pattern of Cu_2_NCN before and after electrolysis. **(b)** IR spectra of Cu_2_NCN before and after electrolysis.


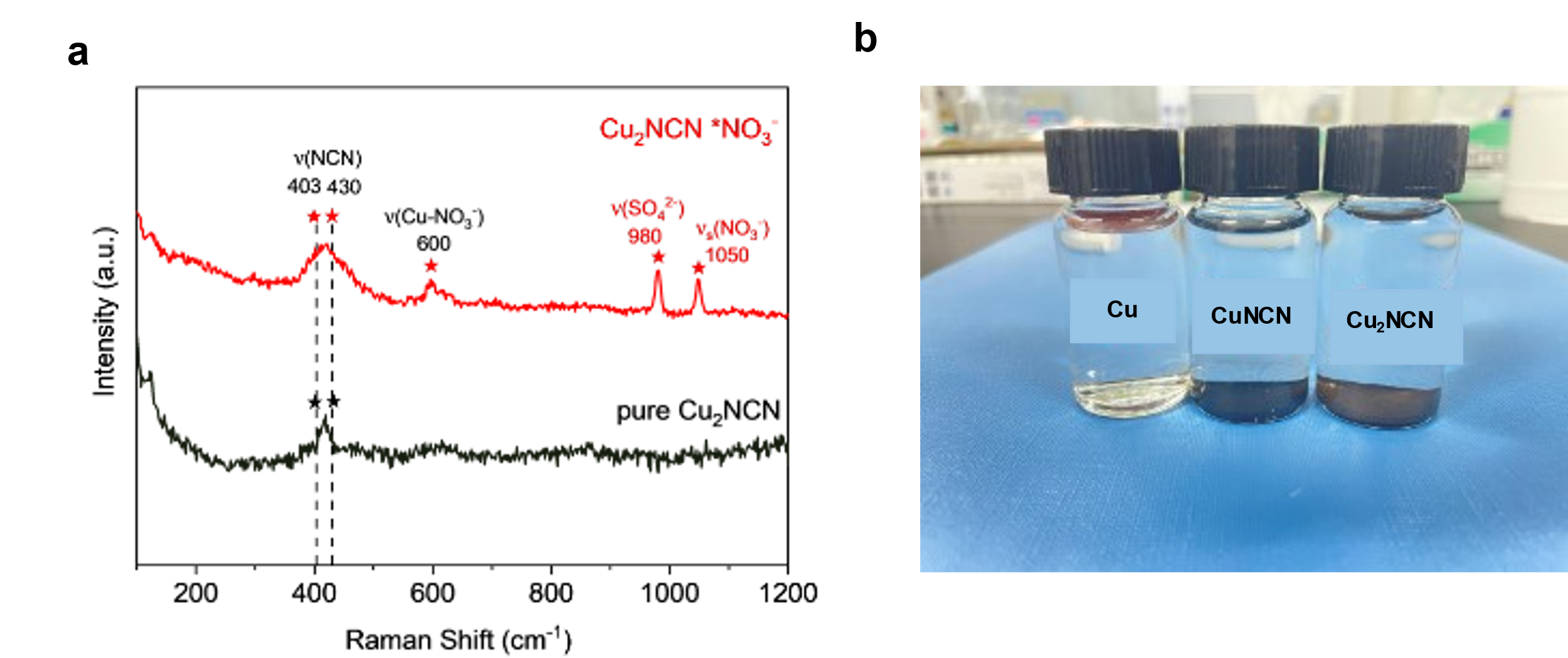


**Supplementary Fig. S13 | Spectroscopic characterization on the adsorption of NO_3_^–^ by Cu_2_NCN.** (a) Cu_2_NCN were physically immersed in NO_3_^–^ contained electrolyte for 24 h. Raman spectra of Cu_2_NCN before and after physical immersion in NO_3_^–^ contained electrolyte. (b) Photograph of immersing in NO_3_^–^ containing electrolyte for 24 h.


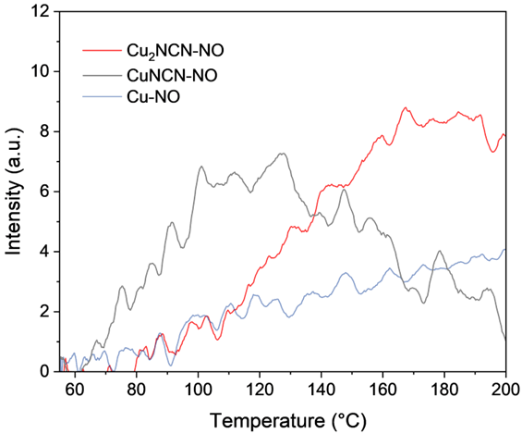


**Supplementary Fig. S14 |** NO-TPD curves of pristine Cu_2_NCN, CuNCN and Cu nanocrystals. Cu_2_NCN exhibits greater adsorption of NO than CuNCN and Cu. The results showed that polarized Cu_2_NCN exhibits the strongest adsorption capacity of NO in comparison to non-polarized CuNCN and Cu.


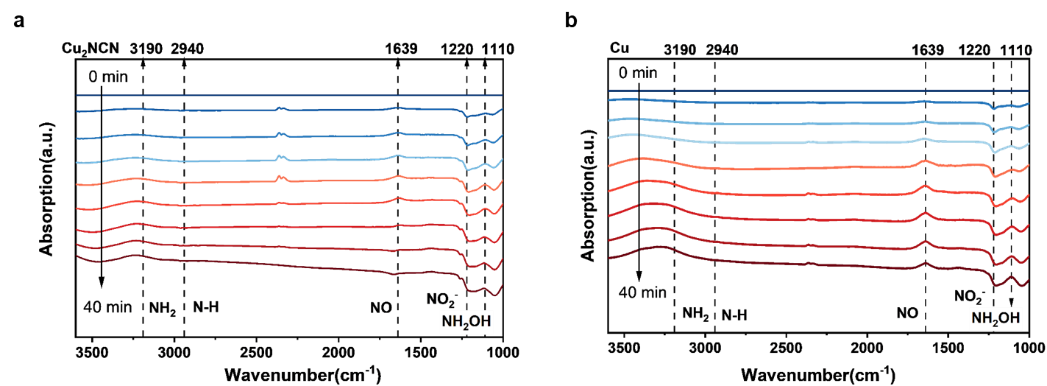


**Supplementary Fig. S15 | In situ FT-IR spectra of Cu_2_NCN (a) and Cu (b)** collected during NO_3_RR under –0.7 V vs. RHE after reaction for different periods (0–40 min).

With the increase of reaction time (0-40 min), the nitrogen oxygen intermediate (NO_x_) on the surface of Cu_2_NCN reacts rapidly to deoxidize and hydrogenate, producing relatively more NH_2_OH. While more NO_2_ and NO intermediates are accumulated on the surface of pure Cu and it is difficult to further react due to the weak interaction between the symmetric copper site and NO_x_.

**
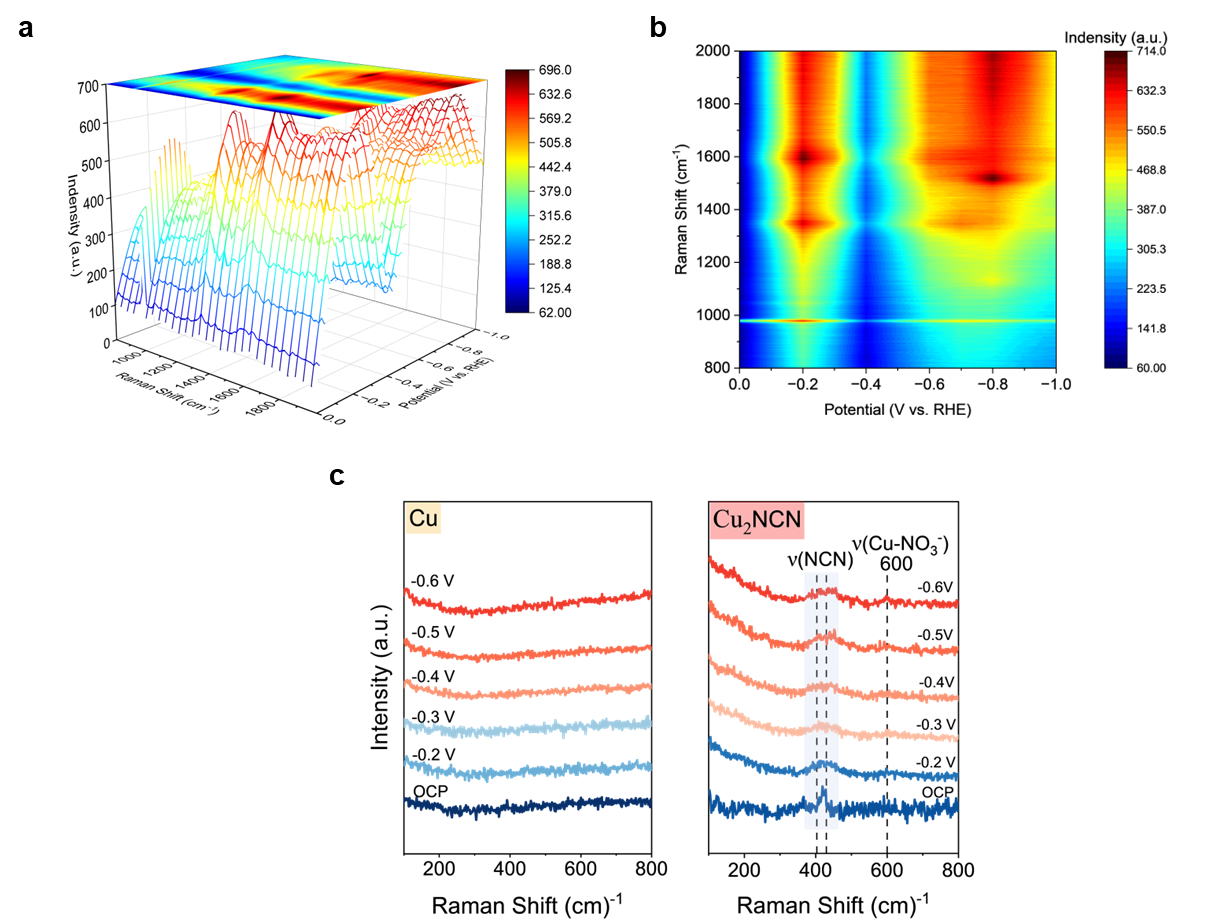
**

**Supplementary Fig. S16 |** **(a, b)** 3D map of the In-situ Raman spectra (800−2000 cm^−1^) of Cu_2_NCN in NO_3_RR at a potential range from OCP to −1.0 V (vs. RHE). **(c)** In-situ Raman spectra (150−800 cm^−1^) of Cu_2_NCN and Cu in NO_3_RR at a potential range from OCP to −0.6 V (vs. RHE). Compared to symmetric Cu, Cu_2_NCN shows the peak of nitrogen oxide adsorption at ~600 cm^−1^ and the peak of [N−C≡N]^2-^ near 400 cm^−1^.


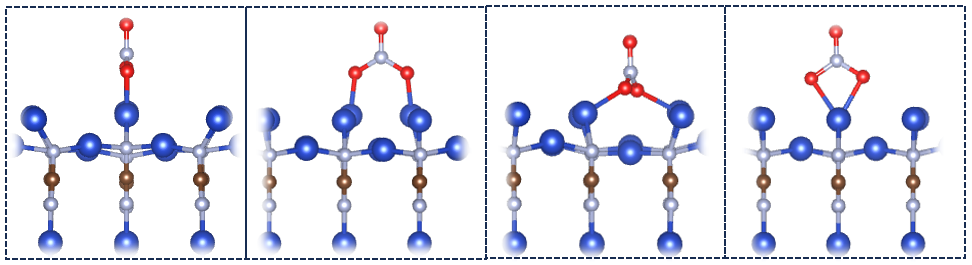


**Supplementary Fig. S17 | Possible optimized configurations of NO_3_* adsorption on Cu_2_NCN(100)** from side view and their corresponding adsorption free energies. Color code: Cu-blue, N-light blue, C-brown and O-red.


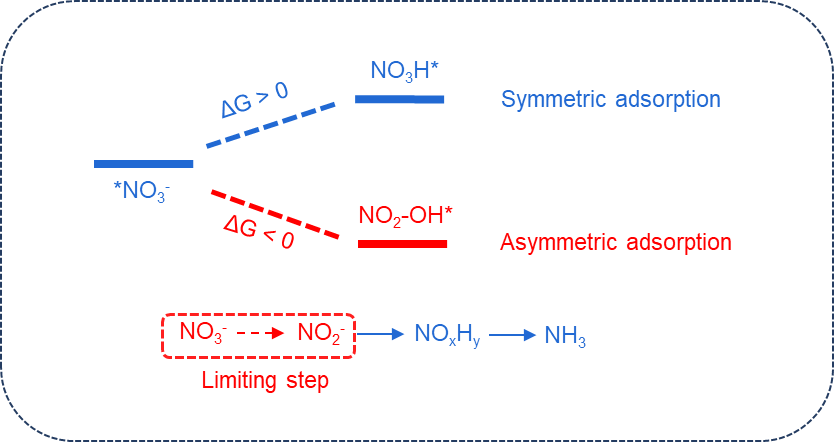


**Supplementary Fig. S18 | Schematic diagram on symmetric and asymmetric adsorption.**


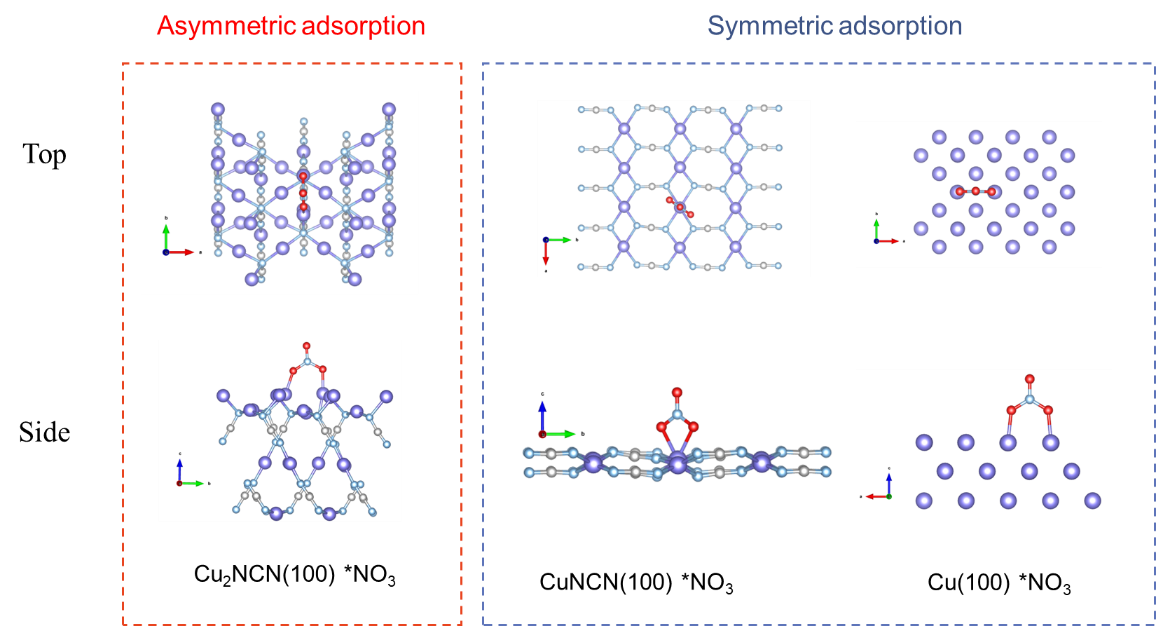


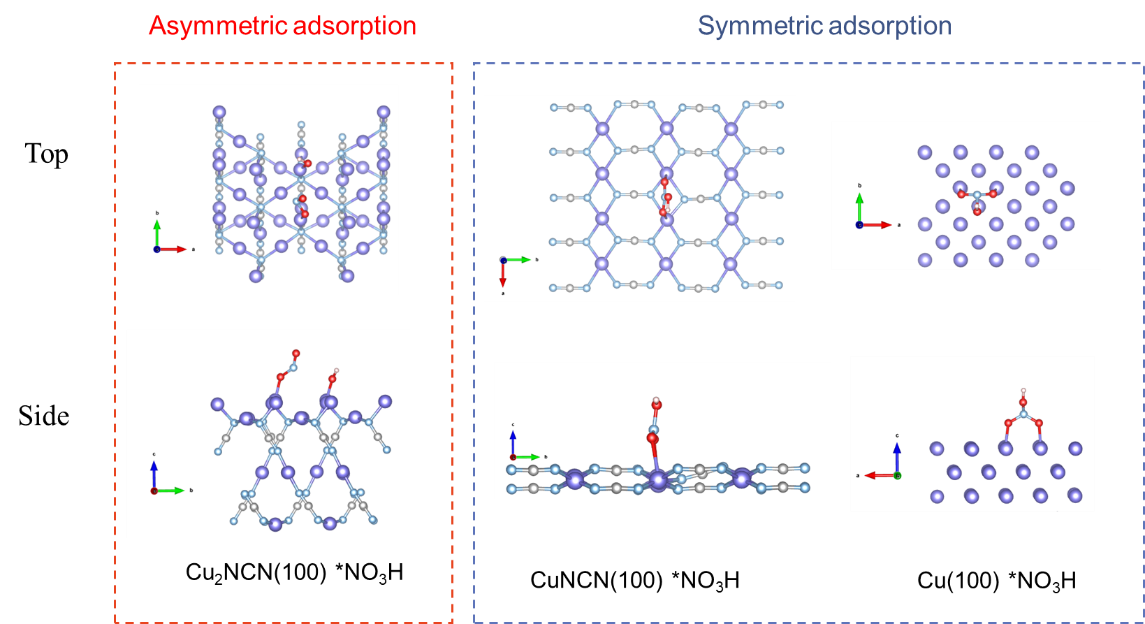


**Supplementary Fig. S19 |** Optimized structures of *NO_3_ and *NO_3_H on Cu_2_NCN, CuNCN and Cu, respectively.


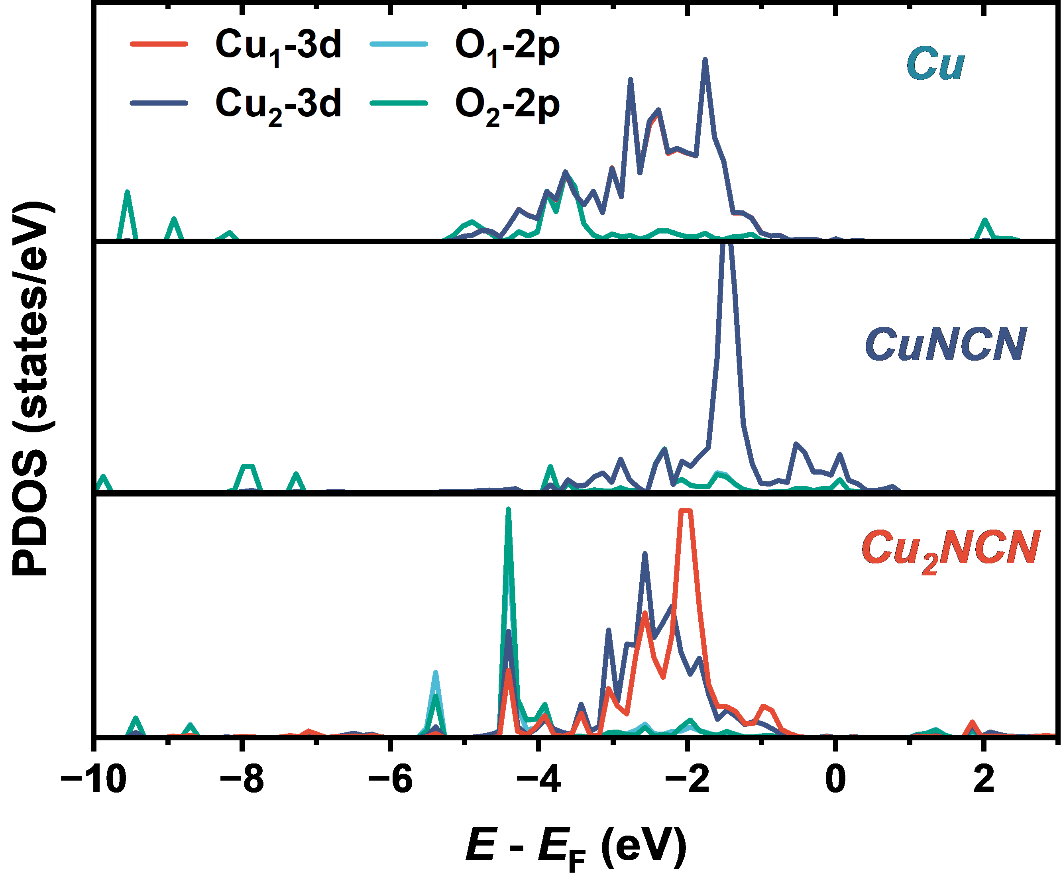


**Supplementary Fig. S20 | Partial density of states (PDOS) analysis** for O-2p orbitals of NO_3_* adsorbed species and Cu-3d orbitals of Cu_2_NCN, CuNCN and Cu surfaces. The Fermi level is set to zero at the dotted line. Note Cu_1_-3d and O_1_-2p orbitals are masked by Cu_2_-3d and O_2_-2p orbitals.


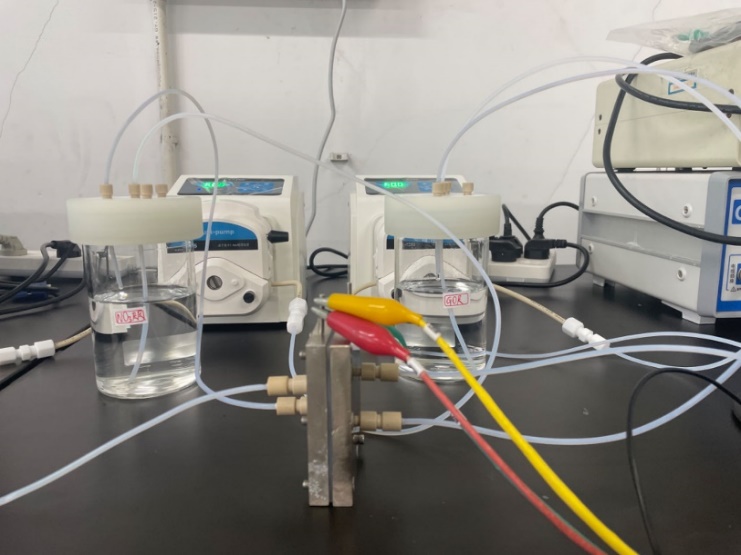


**Supplementary Fig. S21 |** Photographs showing the MEA electrolyzer with a 4 cm^2^ active geometric area of the flow filed on each side. And the second picture exhibits the PER system with Cu_2_NCN as the NO_3_RR cathode and NiCo_2_O_4_ as the GOR anode adopts a neutral electrolyte at the cathode side and an alkaline electrolyte at the anode side for paired electrosynthesis, showing the simultaneous production of value-added ammonia and formic acid.

**Supplementary Tables**

**Supplementary Table S1.** **EXAFS analyses.**

Fitting results (i.e., as-acquired values of structural parameters) for the first coordination shell (1 ‒ 3 Å) of Cu Atoms in Cu_2_NCN and CuNCN samples from Cu K-edge EXAFS Data. (ΔE0: energy shift; N: coordination number; ⟨R⟩: interatomic distance; σ^2^: Debye-Waller factor).

| **Sample** | **Fit path** | **CN** | **ΔE (eV)** | **R (Å)** | **σ^2^ (Å^2^)** | **R factor** |
| --- | --- | --- | --- | --- | --- | --- |
| CuNCN | Cu-N | 3.9±0.9 | 6.4±2.3 | 1.988±0.022 | 0.003±0.002 | 0.02 |
| Cu_2_NCN | Cu-N | 2.1±0.6 | 6.3±3.1 | 1.901±0.031 | 0.004±0.002 | 0.05 |

Note: EXAFS is not able to distinguish bond length less than 0.1 A difference. The bond length of one sample represents an average value. The amplitude reduction factor is set to be 0.75.

**Supplementary Table S2**. **Comparison of onset potential and price of NH_3_ between this work and recent reports.**

| **Catalysts** | **Onset Potential/V** | **Price of NH_3_/ $ kg^-1^** | **Ref.** |
| --- | --- | --- | --- |
| Ru_15_Co_85_ HNDs | 0.4 | 0.49 | ^[4]^ |
| Co array | 0.11 | 0.69 | ^[5]^ |
| O–Cu–PTCDA | –0.2 | 0.82 | ^[6]^ |
| Cu/Cu_2_O NWAs | –0.55 | 0.93 | ^[7]^ |
| TiO_2-X_ | –0.84 | 1.07 | ^[8]^ |
| Fe-PPy SACs | 0 | 0.83 | ^[9]^ |
| PdCu | –0.4 | 0.93 | ^[10]^ |
| Cu nanosheets | –0.15 | 0.61 | ^[11]^ |
| CoP NAs/CFC | –0.1 | 0.69 | ^[12]^ |
| Cu_50_Ni_50_ alloy | 0 | 0.62 | ^[13]^ |
| RuCu | 0.216 | 0.58 | ^[14]^ |
| Bi | −0.2 | 0.84 | ^[15]^ |
| Co/Cu based | 0.025 | 0.70 | ^[16]^ |
| PdCu | −0.2 | 0.83 | ^[17]^ |
| PtRu | 0.1 | 0.54 | ^[18]^ |
| **Cu_2_NCN** | **0.5** | **0.38** | **This work** |

Considering the upcoming industrialization of the NO_3_RR ^[19]^, the cost of producing ammonia is significant, especially compared to commercial ammonia from the Haber-Bosch process. Notably, this is only a simple cost accounting based on electricity price without considering capital costs and ohmic losses, etc. However, it is an important parameter to compare the NO_3_RR performance of different catalysts. The production cost per kilo ammonia over Cu_2_NCN is calculated as $ 0.38, lower than the commercial price from the Haber-Bosch process ($1.0~1.5, sold in the USA) ^[20]^.

**Supplementary Table S3**. **NO_3_RR performance comparison for various electrocatalysts.**

| **Catalysts** | **Electrolyte** | **FE_NH3_(%)** | **Potential**  **(V vs. RHE)** | **NH_3_ yield rate**  **(mg h^-1^ cm^-2^)** | **Current**  **(mA cm^-2^)** | **Ref.** |
| --- | --- | --- | --- | --- | --- | --- |
| **Cu_2_NCN** | **500 ppm NO_3_^–^–N,**  **0.5 M K_2_SO_4_** | **99** | **–0.7** | **17** | **150** | **This work** |
| Cu/Cu_2_O NWAs | 200 ppm NO_3_^–^–N,  0.5 M Na_2_SO_4_ | 95.8 | **–**0.85 | 4.08 | 105 | ^[7]^ |
| O–Cu–PTCDA | 500 ppm NO_3_^–^,  0.1 M PBS | 83.5 | **–**1.0 | 0.436 | 40 | ^[6]^ |
| CuCl_BEF | 100 mg L^–1^ NO_3_^–^,  0.5 M Na_2_SO_4_ | 44.7 | **–**1.0 | 1.82 | 60 | ^[21]^ |
| Cu(B) | 100 ppm NO_3_^–^–N,  0.5 M K_2_SO_4_,  0.1 M PBS | ~100 | **–**0.6 | 1.05 | 75 | ^[22]^ |
| Cu–cis–N_2_O_2_ | 1000 ppm NO_3_^–^–N,  0.5 M Na_2_SO_4_ | ~84 | **–**1.0 | 27.84 | 80 | ^[23]^ |
| RuFe-FeNC | 0.1 M NO_3_^–^,  0.5 M K_2_SO_4_ | 92.2 | **–**1.4 | 0.012 | 120 | ^[24]^ |
| Fe/Ni_2_P | 50 mM NO_3_^–^,  0.2 M K_2_SO_4_ | 94.3 | –0.4 | 4.16 | 170 | ^[25]^ |
| Fe SAC | 0.5 M NO_3_^–^,  0.1 M K_2_SO_4_ | 74.9 | –0.66 | ~ 2.4 | 125 | ^[26]^ |
| Co/CoO NSAS | 200 ppm NO_3_^–^–N,  0.1 M Na_2_SO_4_ | ~93.8 | ~ –0.64 | 3.30 | 80 | ^[27]^ |
| TiO_2-X_ | 50 ppm NO_3_^–^–N,  0.5 M Na_2_SO_4_ | 95.2 | ~ –0.94 | 0.765 | 12 | ^[8]^ |
| CuPc@  MXene | 50 mg/L NO_3_^–^–N,  0.5 M Na_2_SO_4_ | N.A. | ~ –1.06 | 0.72 | 40 | ^[28]^ |
| Co_3_O_4_-TiO_2_/Ti | 50 mg/L NO_3_^–^,  0.1 M Na_2_SO_4_ | N.A. | ~ –0.8 | N.A. | 16 | ^[29]^ |
| Co-Fe@Fe_2_O_3_ | 500 ppm NO_3_^–^–N,  0.1 M Na_2_SO_4_ | 85.2 | –0.745 | 0.88 | 25 | ^[30]^ |
| Cu SAAs | 20 mM NO_3_^–^,  0.1 M PBS | 77.7 | –0.8 | 0.53 | 20 | ^[31]^ |

**Supplementary Table S4. Theoretical calculation of adsorption of *NO_3_ on Cu_2_NCN.**

| **THz** | **2PiTHz** | **cm^–1^** | **meV** |
| --- | --- | --- | --- |
| 1 f = 43.1177 | 270.9166 | 1438.2524 | 178.3205 |
| 2 f = 32.2437 | 202.5932 | 1075.5343 | 133.3492 |
| 3 f = 27.3911 | 172.1035 | 913.6692 | 113.2805 |
| 4 f = 22.1955 | 139.4581 | 740.3606 | 91.7930 |
| 5 f = 20.5459 | 129.0935 | 685.3365 | 84.9708 |
| 6 f = 19.4595 | 122.2674 | 649.0978 | 80.4778 |
| 7 f = 8.8270 | 55.4614 | 294.4358 | 36.5054 |

Note: The adsorption peak of NO_3_^–^ near 1050 cm**^–1^** has been confirmed by experiments, indicating the vibration peak of nitrate near 600 cm**^–1^** was calculated and matched with Raman.

**Supplementary Table S5. Free energy of various intermediates on three catalysts, setting the first adsorbed state as the reference level**.

| **Sample**  **Intermediates** | **Cu_2_NCN** | **Cu** | **CuNCN** |
| --- | --- | --- | --- |
| * + NO_3_^−^ | 0.00 | 0.00 | 0.00 |
| NO_3_* | −3.2 | −0.16 | 0.89 |
| NO_3_H* | −4.09 | 0.52 | 0.20 |
| NO_2_* | −4.22 | −2.06 | -1.03 |
| NO_2_H* | −4.27 | −1.60 | -1.59 |
| * + NO_2_ | −0.62 | −0.62 | -0.62 |
| NO* | −4.76 | −3.18 | -2.74 |
| NHO* | −5.11 | −3.11 | -2.52 |
| NOH* | −4.95 | −2.89 | -2.81 |
| * + NO | −2.22 | −2.22 | -2.23 |
| NHOH* | −5.59 | −3.37 | -3.29 |
| NH_2_O* | −6.09 | −3.81 | -2.72 |
| N* | −6.08 | −3.75 | -3.57 |
| NH_2_OH* | −7.48 | −3.90 | -3.69 |
| NH* | −6.97 | −4.90 | -4.65 |
| NH_2_* | −8.21 | −6.19 | -5.53 |
| NH_3_* | -7.96 | −6.65 | -6.71 |
| * + NH_3_ | -6.29 | −6.29 | -6.29 |

**Supplementary Table S6.** **Products distributions for NO_3_RR in MEA-based electrolyzers at a full-cell voltage of 2.4 V.**

| **Product** | **Faradaic efficiency (%)** |
| --- | --- |
| NH_3_ | 94 |
| Formic acid | 96 |

**Supplementary Note 1.**

Theoretical Gibb’s free energy (ΔG) and potential (E) of reaction for the anodic electro-oxidation of glycerol (C_3_H_8_O_3_) to formic acid (HCOOH) coupled to the cathodic HER^[32]^ (Standard molar free energy of formation (△G_f_): C_3_H_8_O_3_ (l): −478.6 KJ mol^−1^, H_2_O (l): −237.13 KJ mol^−1^, OH^−^ (l): −157.244 KJ mol^−1^, HCOOH (l): −361.3 KJ mol^−1^):

Anode reaction: C_3_H_8_O_3_ + 8 OH^−^ − 8e^−^ → 3 HCOOH + 5 H_2_O, E _Anode reaction_ = 0.69 V

**References**

[1] a) M. Jouny, W. Luc, F. Jiao, Ind. Eng. Chem. Res. **2018**, 57, 2165; b) P. De Luna, C. Hahn, D. Higgins, S. A. Jaffer, T. F. Jaramillo, E. H. Sargent, Science **2019**, 364, eaav3506.

[2] X. Han, H. Sheng, C. Yu, T. W. Walker, G. W. Huber, J. Qiu, S. Jin, ACS Cataly. **2020**, 10, 6741.

[3] J. Halldin Stenlid, A. J. Johansson, T. Brinck, Phys. Chem. Chem. Phys. **2018**, 20, 2676.

[4] S. Han, H. Li, T. Li, F. Chen, R. Yang, Y. Yu, B. Zhang, Nat. Catal. **2023**, 6, 402.

[5] X. Deng, Yang, Y., Wang, L., Fu, X. Z. & Luo, J. L., Adv. Sci. **2021**, 8, 2004523.

[6] G.-F. Chen, Y. Yuan, H. Jiang, S.-Y. Ren, L.-X. Ding, L. Ma, T. Wu, J. Lu, H. Wang, Nat. Energy **2020**, 5, 605.

[7] Y. Wang, W. Zhou, R. Jia, Y. Yu, B. Zhang, Angew. Chem. Int. Ed. **2020**, 59, 5350.

[8] R. Jia, Y. Wang, C. Wang, Y. Ling, Y. Yu, B. Zhang, ACS Cataly. **2020**, 10, 3533.

[9] P. Li, Z. Jin, Z. Fang, G. Yu, Energy Environ. Sci. **2021**, 14, 3522.

[10] H. Yin, Y. Peng, S. Xiong, J. Chen, C. Wang, R. Wang, Z. Chen, Y. Kuwahara, J. Luo, H. Yamashita, J. Li, Chem Catal. **2020**, 1, 1088.

[11] X. Fu, X. Zhao, X. Hu, K. He, Y. Yu, T. Li, Q. Tu, X. Qian, Q. Yue, M. R. Wasielewski, Y. Kang, Appl. Mater. Today **2020**, 19, 100620.

[12] S. Ye, Z. Chen, G. Zhang, W. Chen, C. Peng, X. Yang, L. Zheng, Y. Li, X. Ren, H. Cao, D. Xue, J. Qiu, Q. Zhang, J. Liu, Energy Environ. Sci. **2022**, 15, 760.

[13] Y. Wang, A. Xu, Z. Wang, L. Huang, J. Li, F. Li, J. Wicks, M. Luo, D.-H. Nam, C.-S. Tan, Y. Ding, J. Wu, Y. Lum, C.-T. Dinh, D. Sinton, G. Zheng, E. H. Sargent, J. Am. Chem. Soc. **2020**, 142, 5702.

[14] F.-Y. Chen, Z.-Y. Wu, S. Gupta, D. J. Rivera, S. V. Lambeets, S. Pecaut, J. Y. T. Kim, P. Zhu, Y. Z. Finfrock, D. M. Meira, G. King, G. Gao, W. Xu, D. A. Cullen, H. Zhou, Y. Han, D. E. Perea, C. L. Muhich, H. Wang, Nat. Nanotechnol. **2022**, 17, 759.

[15] N. Zhang, J. Shang, X. Deng, L. Cai, R. Long, Y. Xiong, Y. Chai, ACS Nano **2022**, 16, 4795.

[16] W. He, J. Zhang, S. Dieckhöfer, S. Varhade, A. C. Brix, A. Lielpetere, S. Seisel, J. R. C. Junqueira, W. Schuhmann, Nat. Commun. **2022**, 13, 1129.

[17] J. Gao, N. Shi, Y. Li, B. Jiang, T. Marhaba, W. Zhang, Environ. Sci. Technol. **2022**, 56, 11602.

[18] Z. Wang, S. D. Young, B. R. Goldsmith, N. Singh, J. Catal. **2021**, 395, 143.

[19] a) G. F. Chen, Y. Yuan, H. Jiang, S. Y. Ren, H. Wang, Nat. Energy **2020**, 5, 1; b) W. Zheng, L. Zhu, Z. Yan, Z. Lin, Z. Lei, Y. Zhang, H. Xu, Z. Dang, C. Wei, C. Feng, Environ. Sci. Technol. **2021**, 55, 13231.

[20] D. R. MacFarlane, P. V. Cherepanov, J. Choi, B. H. R. Suryanto, R. Y. Hodgetts, J. M. Bakker, F. M. Ferrero Vallana, A. N. Simonov, Joule **2020**, 4, 1186.

[21] W. J. Sun, H. Q. Ji, L. X. Li, H. Y. Zhang, Z. K. Wang, J. H. He, J. M. Lu, Angew. Chem. Int. Ed. **2021**, 60, 22933.

[22] L. H. Zhang, Y. Jia, J. Zhan, G. Liu, G. Liu, F. Li, F. Yu, Angew. Chem. Int. Ed. **2023**, 62, e202303483.

[23] X. F. Cheng, J. H. He, H. Q. Ji, H. Y. Zhang, Q. Cao, W. J. Sun, C. L. Yan, J. M. Lu, Adv. Mater. **2022**, 34, 2205767.

[24] X. Zhao, Y. Jiang, M. Wang, S. Liu, Z. Wang, T. Qian, C. Yan, Adv. Energy Mater. **2023**, 13, 2301409.

[25] R. Zhang, Y. Guo, S. Zhang, D. Chen, Y. Zhao, Z. Huang, L. Ma, P. Li, Q. Yang, G. Liang, C. Zhi, Adv. Energy Mater. **2022**, 12, 2103872.

[26] Z. Y. Wu, M. Karamad, X. Yong, Q. Huang, D. A. Cullen, P. Zhu, C. Xia, Q. Xiao, M. Shakouri, F. Y. Chen, J. Y. T. Kim, Y. Xia, K. Heck, Y. Hu, M. S. Wong, Q. Li, I. Gates, S. Siahrostami, H. Wang, Nat. Commun. **2021**, 12, 2870.

[27] Y.-Q. Yu, C. Wang, Y. Yu, Y. Wang, B. Zhang, Sci. China Chem. **2020**, 63, 1469

[28] L.-X. Li, W.-J. Sun, H.-Y. Zhang, J.-L. Wei, S.-X. Wang, J.-H. He, N.-J. Li, Q.-F. Xu, D.-Y. Chen, H. Li, J.-M. Lu, J. Mater. Chem. A **2021**, 9, 21771.

[29] J. Gao, B. Jiang, C. Ni, Y.-f. Qi, Y. Zhang, N. Oturan, M. A. Oturan, Appl. Catal. B Environ. **2019**, 254, 391.

[30] S. Zhang, M. Li, J. Li, Q. Song, X. Liu, Proc. Natl. Acad. Sci. **2022**, 119, e2115504119.

[31] P. Li, R. Li, Y. Liu, M. Xie, Z. Jin, G. Yu, J. Am. Chem. Soc. **2023**, 145, 6471.

[32] a) Y. Li, X. Wei, L. Chen, J. Shi, M. He, Nature Communications **2019**, 10; b) S. Li, P. Ma, C. Gao, L. Liu, X. Wang, M. Shakouri, R. Chernikov, K. Wang, D. Liu, R. Ma, J. Wang, Energy Environ. Sci. **2022**, 15, 3004.
